# Trends in Racial and Ethnic Disparities in Barriers to Timely Medical Care Among Adults in the US, 1999 to 2018

**DOI:** 10.1001/jamahealthforum.2022.3856

**Published:** 2022-10-28

**Authors:** César Caraballo, Chima D. Ndumele, Brita Roy, Yuan Lu, Carley Riley, Jeph Herrin, Harlan M. Krumholz

**Affiliations:** 1 Center for Outcomes Research and Evaluation, Yale New Haven Hospital, New Haven, Connecticut; 2 Section of Cardiovascular Medicine, Department of Internal Medicine, Yale School of Medicine, New Haven, Connecticut; 3 Department of Health Policy and Management, Yale School of Public Health, New Haven, Connecticut; 4 Section of General Internal Medicine, Department of Internal Medicine, Yale School of Medicine, New Haven, Connecticut; 5 Department of Chronic Disease Epidemiology, Yale School of Public Health, New Haven, Connecticut; 6 Department of Pediatrics, University of Cincinnati College of Medicine, Cincinnati, Ohio; 7 Division of Critical Care Medicine, Cincinnati Children’s Hospital Medical Center, Cincinnati, Ohio

## Abstract

**Importance:**

Racial and ethnic disparities in delayed medical care for reasons that are not directly associated with the cost of care remain understudied.

**Objective:**

To describe trends in racial and ethnic disparities in barriers to timely medical care among adults during a recent 20-year period.

**Design, Setting, and Participants:**

This was a serial cross-sectional study of 590 603 noninstitutionalized adults in the US using data from the National Health Interview Survey from 1999 to 2018. Data analyses were performed from December 2021 through August 2022.

**Exposures:**

Self-reported race, ethnicity, household income, and sex.

**Main Outcomes and Measures:**

Temporal trends in disparities regarding 5 specific barriers to timely medical care: inability to get through by telephone, no appointment available soon enough, long waiting times, inconvenient office or clinic hours, and lack of transportation.

**Results:**

The study cohort comprised 590 603 adult respondents (mean [SE] age, 46.00 [0.07] years; 329 638 [51.9%] female; 27 447 [4.7%] Asian, 83 929 [11.8%] Black, 98 692 [13.8%] Hispanic/Latino, and 380 535 [69.7%] White). In 1999, the proportion of each race and ethnicity group reporting any of the 5 barriers to timely medical care was 7.3% among the Asian group; 6.9%, Black; 7.9%, Hispanic/Latino; and 7.0%, White (*P* > .05 for each difference compared with White individuals). From 1999 to 2018, this proportion increased across all 4 race and ethnicity groups (by 5.7, 8.0, 8.1, and 5.9 percentage points [pp] among Asian, Black, Hispanic/Latino, and White individuals, respectively; *P* < .001 for each), slightly increasing the disparities between groups. In 2018, compared with White individuals, the proportion reporting any barrier was 2.1 and 3.1 pp higher among Black and Hispanic/Latino individuals (*P* = .03 and *P* = .001, respectively). There was no significant difference in prevalence between Asian and White individuals. There was a significant increase in the difference in prevalence between Black individuals and White individuals who reported delaying care because of long waiting times at the clinic or medical office and because of a lack of transportation (1.5 pp and 1.8 pp; *P* = .03 and *P* = .01, respectively). In addition, the difference in prevalence between Hispanic/Latino and White individuals who reported delaying care because of long waiting times increased significantly (2.6 pp; *P* < .001).

**Conclusions and Relevance:**

The findings of this serial cross-sectional study of data from the National Health Interview Survey suggest that barriers to timely medical care in the US increased for all population groups from 1999 to 2018, with associated increases in disparities among race and ethnicity groups. Interventions beyond those currently implemented are needed to improve access to medical care and to eliminate disparities among race and ethnicity groups.

## Key Points

**Question:**

How have trends in racial and ethnic disparities in barriers to timely medical care among adults in the US changed from 1999 to 2018?

**Findings:**

This serial cross-sectional study including 590 603 adults in the US found that the prevalence of 5 barriers to timely medical care that are not directly related to cost of care increased across all race and ethnicity groups. By 2018, barrier prevalence was significantly higher among Black and Hispanic/Latino individuals who were more likely than White individuals to report experiencing long waiting times and lack of transportation.

**Meaning:**

The findings of this serial cross-sectional study suggest that the marked differences among race and ethnicity groups and barriers to timely medical care that are not directly cost related may be contributing to health disparities.

## Teaser

This large serial cross-sectional study of US adults uses data from the National Health Interview Survey (1999-2018) to examine racial and ethnic trends in 5 barriers to timely medical care that are not directly related to cost of care.

## Introduction

There are racial and ethnic disparities in access to health care in the US^1,2,3,4^ despite national efforts to eliminate them.^5,6,7^ For example, compared with White individuals, Black and Latino individuals persistently had a higher prevalence of lack of health insurance and cost-related unmet medical needs from 1999 to 2018.^4^ Race and ethnicity reflect cultural identity. Race- and ethnicity-based injustices have produced unequal and asymmetric supply-side access to delivery systems and information. There has been considerable focus on the need to remove barriers to coverage and affordability; however, less attention has been focused on barriers that are not directly related to the cost of care and that may disproportionally affect patients of racial and ethnic minority groups who may have greater social risk factors.

Black, Latino, and low-income individuals are more likely to experience barriers to timely medical care that are not directly related to cost of care,^8,9,10,11,12^ such as long waiting times at the physician’s office, inconvenient office hours, and lack of transportation. Importantly, in the years after the Affordable Care Act (ACA) was implemented, there was an increase in overall appointment availability and health care utilization but no improvement in waiting times.^13,14,15,16^ However, despite overall progress with some indicators, it is not known how racial and ethnic disparities in these barriers to timely medical care have changed during the past decades at the national level—and whether there has been any progress in eliminating them.

Accordingly, to comprehensively assess the nation’s performance on these indicators during the past 2 decades, we used data from the National Health Interview Survey (NHIS) to describe trends in racial and ethnic disparities in barriers to timely medical care that are not directly cost related from 1999 to 2018. Given that there are differences in these barriers by sex and income,^10,17,18^ we also stratified the main findings by sex and income level. This report aims to inform on trends in racial and ethnic disparities regarding 5 specific barriers to timely medical care that are not directly related to cost of care. It is important to note that although we are studying these specific barriers separately from indicators of insurance coverage and affordability of care, the complexity of the US health care system makes it implausible to isolate these from an individual’s health insurance status and overall financial situation. Thus, as a sensitivity analysis, we also stratified the main analysis by insurance status and presence of cost-related barriers.

## Methods

This serial cross-sectional study was reviewed and approved by the institutional review board of Yale University. Informed consent was waived because we used only deidentified publicly available data. The study follows the Strengthening the Reporting of Observational Studies in Epidemiology (STROBE) reporting guideline.

### Data Source

In this serial cross-sectional study, we used data from the annual NHIS, from 1999 to 2018, obtained from the Integrated Public Use Microdata Series Health Surveys.^19^ The NHIS is a series of annual cross-sectional national surveys that provide information on the health of the noninstitutionalized population of the US. The sample design uses a multistage area probability design that adjusts for nonresponse and further allows for a national representative sampling of households and individuals, including traditionally underrepresented groups.^20^ The survey consists of a questionnaire divided into 4 cores (eMethods in the Supplement). In this study, we used data from the Sample Adult Core files, with a mean conditional response rate and final response rate during the study period of 81% and 64.8%, respectively.

### Study Population

Of 603 028 adults (≥18 years old) interviewed in the NHIS from 1999 to 2018, we excluded 5752 because their records were missing data on barriers to timely medical care that are not directly related to cost of care. Because of small numbers, we excluded 6673 respondents who self-reported as non-Hispanic Alaska Native, non-Hispanic American Indian, non-Hispanic with no race, or “other” race (additional details available in eFigure 1 and eMethods in the Supplement).

### Demographic Variables

In the NHIS, Hispanic or Latino ethnicity was ascertained by the question, “Do you consider yourself Latino/Hispanic?” Race was ascertained by the question, “What race do you consider yourself to be?” and, if more than 1 was reported, “Which one of these groups would you say best represents your race?” Based on these questions, we classified respondents into 4 mutually exclusive groups by self-reported race and ethnicity (details in the eMethods in the Supplement): non-Hispanic Asian (Asian), non-Hispanic Black or African American (Black), Hispanic or Latino (Hispanic/Latino), and non-Hispanic White (White).

Also from the NHIS, we obtained data on respondents’ age, sex, US geographic region (Northeast, North Central/Midwest, South, West), and self-reported household income level. Based on the household income level relative to the respective year’s federal poverty level (FPL) from the US Census Bureau, income level was categorized as low (<200% of FPL) or middle to high (≥200% of FPL).^4,21,22^

Other respondent characteristics used to stratify the population were insurance status and presence of cost-related barriers to care. Individuals were classified as *uninsured* if at the time of the interview, they reported not having a private health insurance plan, Medicare, Medicaid, military plan, a government- or state-sponsored health plan, or if they had only Indian Health Service coverage. The presence of cost-related barriers to care was defined as answering *yes* to any of these 3 questions: During the past 12 months, (1) “…has medical care been delayed for you because of worry about the cost?”; (2) “…was there any time when you needed medical care, but didn’t get it because you couldn’t afford it?”; and (3) “…was there any time when you needed a prescription medicine but didn’t get it because you couldn’t afford it?”

### Barriers Not Directly Related to Cost

Consistent with previous research,^10^ we defined the presence of barriers not directly related to the cost of care as a response of *yes* to any of the questions. In the past 12 months, did you delay care because:

1. you couldn’t get an appointment soon enough?
2. the (clinic/doctor’s) office wasn’t open when you could get there?
3. you couldn’t get through on the telephone?
4. once you get there, you have to wait too long to see the doctor?
5. you didn’t have transportation?

We also determined the presence of each of these 5 barriers separately.

### Statistical Analysis

To estimate the annual prevalence of each of the barriers to timely medical care, we used multivariable logistic regression models, adjusted for age and region (eMethods in the Supplement). We then subtracted the annual prevalence among White respondents from the annual prevalence among the other race and ethnicity groups for that year, calculating standard errors (SE) for the differences. Using these annual prevalence rates and differences, we calculated trends during the study period by fitting weighted linear regression models, modeling time as a linear spline with knots at 2010 and 2014 (eMethods in the Supplement). Each observation was weighted by the inverse square of the SE of the prevalence to account for the varying precision of each estimate over time. Separately, we used a Ζ test to determine the absolute difference between 1999 and 2018 in each barrier prevalence within each race and ethnicity group and the differences among groups.

Then we separately stratified the analysis described, by sex and household income. Because of the high prevalence of missing income data from participant nonresponse, the income-stratified analysis was based on recommendations from the National Center for Health Statistics for multiply imputed data analysis in the NHIS (eMethods in the Supplement).^23^ We also used ordered logistic regression models to estimate the proportion of individuals with 0, 1, 2, 3, or 4 to 5 specific barriers over the years (eMethods in the Supplement). As a sensitivity analysis, we stratified the main analysis by cost-related insurance status and unmet medical needs in the past 12 months.

For all analyses, a 2-sided *P* < .05 was used to determine statistical significance. All analyses were performed using Stata SE, version 17.0 (StataCorp LLC) and incorporated strata and weights to produce nationally representative estimates using the -*svy-* commands for structured survey data. All results were reported with 95% CIs. All person-weights were pooled and divided by the number of years studied, per NHIS guidance.^24^

## Results

### Study Population Characteristics

 The final sample comprised 590 603 adult respondents (mean [SE] age, 46.00 [0.07] years; 329 638 [weighted percentage, 51.9%] females and 260 965 [48.1%] males). Of these, 27 447 individuals identified as Asian (4.7%; 95% CI, 4.5%-4.8%), 83 929 as Black (11.8%; 95% CI, 11.5%-12.1%), 98 692 as Hispanic/Latino (13.8%; 95% CI, 13.5%-14.2%), and 380 535 as White (69.7%; 95% CI, 69.3%-70.2%). Other characteristics of the population are described in Table 1 and eTable 1 in the Supplement.

**Table 1.** Characteristics of the Study Population, by Race and Ethnicity

Characteristic	Race and ethnicity^a^			
	Asian	Black	Hispanic/Latino	White
Respondents, No. (n = 590 603)	27 447	83 929	98 692	380 535
Age, median (range), y	41 (30-55)	42 (29-55)	38 (28-50)	47 (33-61)
Age category, y				
18-39	45.7 (44.7-46.6)	45.5 (44.9-46.1)	54.2 (53.7-54.8)	35.3 (35.0-35.7)
40-64	41.5 (40.7-42.4)	41.7 (41.3-42.2)	36.7 (36.2-37.1)	44.4 (44.1-44.7)
≥65	12.8 (12.2-13.4)	12.8 (12.4-13.2)	9.1 (8.8-9.4)	20.3 (20.0-20.5)
Sex, female	52.4 (51.6-53.1)	55.3 (54.8-55.7)	49.6 (49.1-50.0)	51.7 (51.5-51.9)
US citizenship (n = 589 337)	68.2 (67.1-69.3)	95.3 (95.0-95.6)	64.5 (63.7-65.3)	98.4 (98.4-98.5)
Education level (n = 586 373)				
<High school	9.8 (9.3-10.4)	18.3 (17.8-18.8)	36.9 (36.2-37.5)	10.3 (10.1-10.5)
High school/GED	16.4 (15.7-17.2)	30.6 (30.1-31.1)	26.2 (25.8-26.7)	27.9 (27.6-28.2)
Some college	22.5 (21.7-23.3)	32.7 (32.1-33.3)	24.3 (23.8-24.8)	31.0 (30.8-31.3)
≥Bachelor’s degree	51.3 (50.1-52.5)	18.4 (17.9-19.0)	12.6 (12.2-13.1)	30.8 (30.4-31.2)
Income <200% FPL^b^	28.2 (24.9-31.7)	46.1 (43.9-48.4)	51.4 (49.4-53.5)	23.9 (23.0-24.9)
Uninsured at interview (n = 588 490)	12.9 (12.3-13.5)	18.4 (18.0-18.9)	34.0 (33.3-34.7)	10.5 (10.3-10.7)
Region of residence^c^				
Northeast	20.1 (18.9-21.3)	16.3 (15.5-17.0)	14.0 (13.2-14.8)	19.3 (18.8-19.7)
Midwest	13.2 (12.3-14.2)	17.8 (16.9-18.6)	9.0 (8.3-9.8)	28.2 (27.6-28.8)
South	21.7 (20.4-23.0)	57.9 (56.7-59.1)	36.3 (35.0-37.6)	33.9 (33.3-34.5)
West	45.0 (43.3-46.7)	8.1 (7.7-8.5)	40.7 (39.3-42.1)	18.7 (18.2-19.2
Married/living with partner (n = 588 349)	64.5 (63.6-65.3)	35.2 (34.6-35.7)	54.0 (53.4-54.5)	58.5 (58.2-58.9)
Employment status (n = 589 945)				
Employed/working	65.3 (64.5-66.2)	60.5 (60.0-61.1)	65.4 (64.8-65.9)	62.9 (62.6-63.2)
Not in labor force	30.8 (29.9-31.6)	31.9 (31.3-32.5)	29.3 (28.8-29.8)	34.0 (33.7-34.3)
Unemployed	3.9 (3.7-4.2)	7.6 (7.4-7.9)	5.3 (5.1-5.5)	3.2 (3.1-3.2)
Current smoker	10.2 (9.7-10.7)	19.7 (19.3-20.1)	13.4 (13.0-13.7)	20.6 (20.4-20.9)
BMI ≥30	9.1 (8.6-9.6)	36.3 (35.8-36.8)	29.6 (29.2-30.1)	25.6 (25.4-25.9)
Chronic health conditions				
Asthma	8.0 (7.6-8.5)	13.2 (12.8-13.5)	9.5 (9.3-9.8)	12.1 (12.0-12.3)
Cancer	2.9 (2.7-3.2)	4.0 (3.8-4.1)	2.8 (2.7-2.9)	10.0 (9.9-10.1)
COPD	1.8 (1.6-2.0)	4.7 (4.5-4.9)	2.8 (2.7-2.9)	5.9 (5.8-6.0)
Diabetes	7.2 (6.8-7.6)	11.0 (10.7-11.3)	8.6 (8.3-8.9)	7.7 (7.5-7.8)
Heart disease	5.6 (5.3-6.0)	9.5 (9.3-9.8)	6.2 (6.0-6.4)	13.2 (13.0-13.4)
Hypertension	21.0 (20.3-21.7)	35.0 (34.5-35.6)	20.0 (19.6-20.4)	28.9 (28.7-29.2)
Kidney disease	1.1 (1.0-1.3)	2.2 (2.1-2.4)	1.8 (1.7-1.9)	1.7 (1.7-1.8)
Liver disease	1.4 (1.2-1.6)	1.1 (1.0-1.2)	1.7 (1.6-1.8)	1.4 (1.4-1.5)
Stroke	1.5 (1.4-1.7)	3.4 (3.3-3.6)	1.7 (1.6-1.8)	2.8 (2.7-2.8)

Abbreviations: BMI, body mass index (calculated as weight in kilograms divided by height in meters squared); COPD, chronic obstructive pulmonary disease; FPL, federal poverty level; GED, general equivalency diploma.

^a^ Data are presented as percentage (95% CI) for categorical variables and median (IQR) for continuous variables. All percentages are weighted and unadjusted.

^b^ Annual family income was categorized as either low income or middle to high income per the respective year’s FPL from the US Census Bureau (<200% and ≥200%, respectively). The weighted proportion of individuals with low income was estimated using multiple imputation.

^c^ Based on the US Census Bureau’s recognized region of the housing unit where the survey participant was interviewed.

### Trends in Racial and Ethnic Differences in Barriers

#### Any Barrier

**Figure 1.**
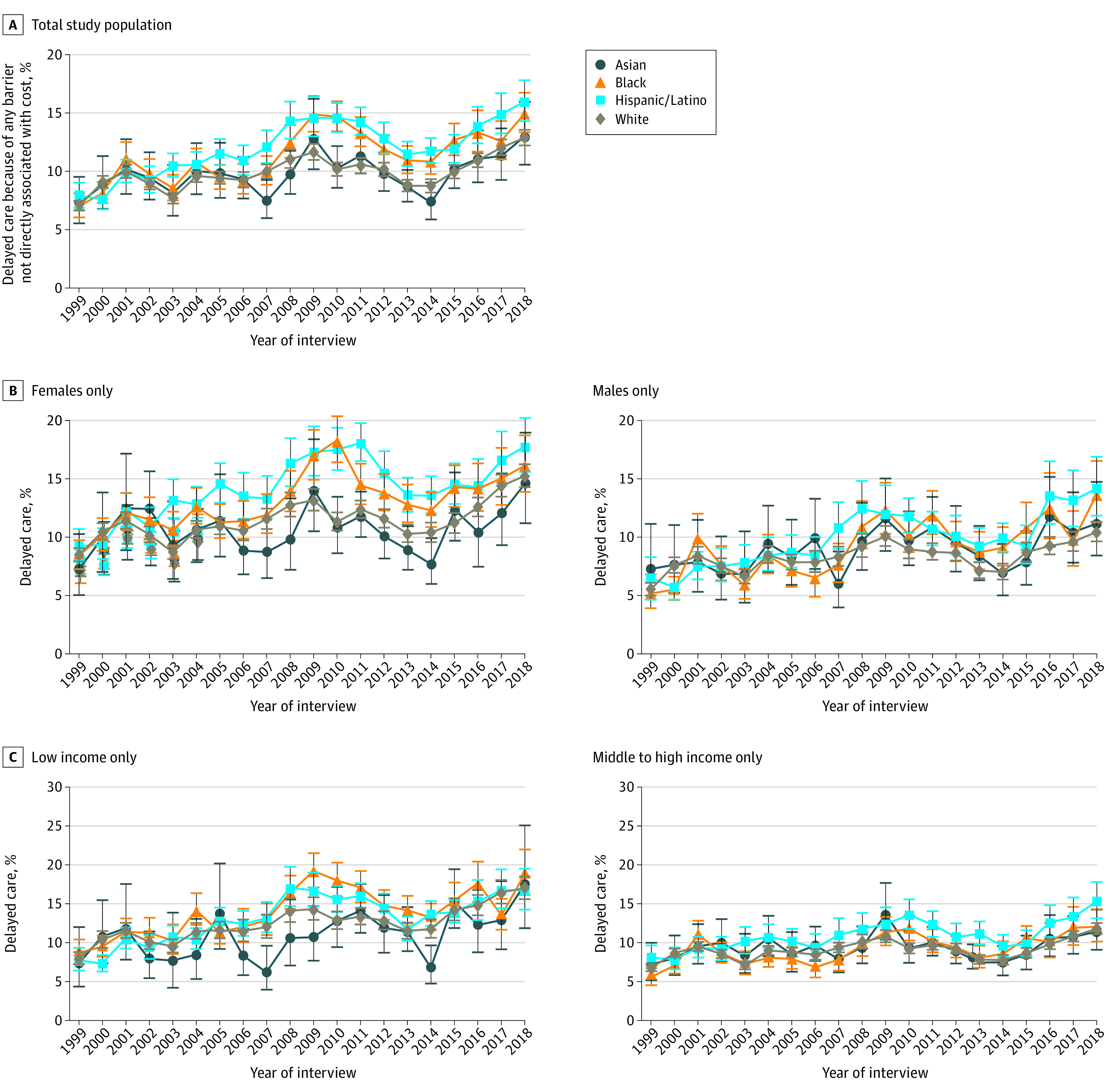
Trends in Annual Prevalence of Barriers to Timely Medical Care Not Directly Related to Cost of Care Among US Adults, by Race and Ethnicity, National Health Interview Survey, 1999 to 2018 Brackets represent 95% CIs. All estimates were adjusted by age and US region.

**Figure 2.**
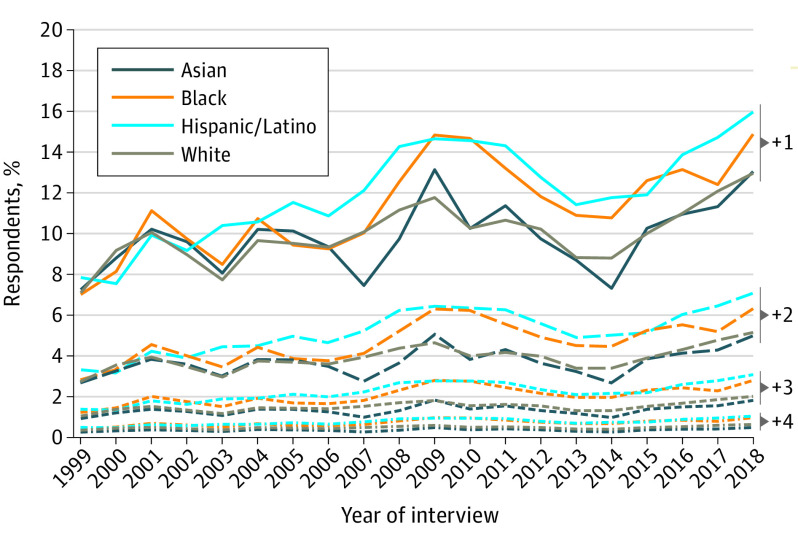
Trends in Estimated Ordered Number of Barriers to Timely Medical Care Not Directly Related to Cost of Care, by Race and Ethnicity, National Health Interview Survey, 1999 to 2018 The ordered number of barriers to care was estimated using ordered logistic regression to estimate the proportion of individuals with 0, 1, 2, 3, or 4 to 5 specific barriers over the years. All estimates were adjusted by age and US region. For visualization purposes, no symbols or brackets are shown. The point estimates data supporting this figure and their respective CIs are available in eTable 6 in the Supplement.

In 1999, the overall proportion of individuals reporting any of the 5 barriers to timely medical care was 7.1% (95% CI, 6.7 to 7.4), and there were no significant differences between White and Asian (+0.2 percentage points [pp]; 95% CI, −1.8 to 2.3; *P* = .83), Hispanic/Latino (+0.9 pp; 95% CI, −0.2 to 2.0; *P* = .12), or Black respondents (−0.03 pp; 95% CI, −1.2 to 1.1; *P* = .95), as shown in Table 2. The adjusted estimated prevalence in 1999 was 7.3% among Asian individuals (95% CI, 5.5% to 9.5%); 6.9% among Black individuals (95% CI, 5.9% to 8.0%); 7.9% among Hispanic/Latino individuals (95% CI, 6.9% to 9.0%); and 7.0% among White individuals (95% CI, 6.6% to 7.5%) (Figure 1). From 1999 to 2018, prevalence increased among all 4 race and ethnicity groups (*P* < .001 for each, as shown in Table 2), slightly increasing the gap between the group of White respondents and the groups of Black and Hispanic/Latino respondents. As reported in eTable 2 in the Supplement, the increase in the difference between these groups occurred mainly between 1999 and 2010 and remained stable thereafter (2011-2018). In 2018, the overall proportion reporting any barrier was 13.5% (95% CI, 12.8% to 14.1%). Compared with the adjusted prevalence among White respondents (12.9%; 95% CI, 12.3% to 13.6%), the proportion was 2.1 pp higher among Black respondents (95% CI, 0.2 to 3.9; *P* = .03) and 3.1 pp higher among Hispanic/Latino respondents (95% CI, 1.2 to 5.0; *P* = .001). There was no significant difference in prevalence between Asian and White respondents (+0.11; 95% CI, −2.68 to 2.90; *P* = .94). Similarly, Black and Hispanic/Latino respondents had the greatest prevalence of 1, 2, 3, or 4 barriers during the study period (Figure 2; eTable 3 in the Supplement).

**Table 2.** Change in the Adjusted Prevalence of Barriers to Timely Medical Care Not Directly Related to Cost of Care, by Race and Ethnicity, National Health Interview Survey, 1999 to 2018

Barrier	Race and ethnicity^a^							
	Asian		Black		Hispanic/Latino		White	
	% (95% CI)	*P* value	% (95% CI)	*P* value	% (95% CI)	*P* value	% (95% CI)	*P* value
**Any barrier**								
Change in prevalence, 1999-2018	+5.74 (+2.38 to +9.11)	< .001	+7.95 (+5.94 to +9.95)	< .001	+8.07 (+6.03 to +10.11)	< .001	+5.85 (+5.06 to +6.63)	< .001
Prevalence in 1999	7.26 (5.55 to 9.53)	NA	7.01 (6.04 to 8.11)	NA	7.93 (6.97 to 9.02)	NA	7.04 (6.64 to 7.47)	NA
Prevalence in 2018	13.01 (10.58 to 15.95)	NA	14.96 (13.35 to 16.75)	NA	15.99 (14.31 to 17.83)	NA	12.89 (12.23 to 13.57)	NA
Change in difference with White individuals, 1999-2018	−0.10 (−3.56 to +3.35)	.95	+2.10 (−0.05 to +4.25)	.06	+2.22 (+0.03 to +4.40)	.05	NA	NA
Difference in 1999	+0.22 (−1.82 to +2.25)	.83	−0.03 (−1.15 to +1.08)	.95	+0.88 (−0.23 to +1.99)	.12	NA	NA
Difference in 2018	+0.11 (−2.68 to +2.90)	.94	+2.06 (+0.22 to +3.91)	.03	+3.10 (+1.22 to +4.98)	.001	NA	NA
**Specific barriers (reported reason for delay of medical care)**								
Couldn’t get through by telephone								
Change in prevalence, 1999-2018	+0.69 (−0.93 to +2.32)	.40	+1.43 (+0.53 to +2.33)	.002	+1.67 (+0.71 to +2.64)	< .001	+0.89 (+0.48 to +1.29)	< .001
Prevalence in 1999	2.17 (1.26 to 3.75)	NA	1.59 (1.24 to 2.04)	NA	1.78 (1.38 to 2.30)	NA	2.18 (1.95 to 2.43)	NA
Prevalence in 2018	2.86 (2.01 to 4.10)	NA	3.02 (2.34 to 3.92)	NA	3.46 (2.71 to 4.41)	NA	3.06 (2.75 to 3.40)	NA
Change in difference with White individuals, 1999-2018	−0.19 (−1.87 to +1.48)	.82	+0.54 (−0.45 to +1.53)	.29	+0.79 (−0.26 to +1.83)	.14	NA	NA
Difference in 1999	−0.01 (−1.28 to +1.27)	.99	−0.58 (−1.05 to −0.11)	.02	−0.39 (−0.91 to +0.12)	.14	NA	NA
Difference in 2018	−0.20 (−1.29 to +0.89)	.72	−0.04 (−0.91 to +0.82)	.92	+0.39 (−0.51 to +1.30)	.40	NA	NA
No appointment soon enough								
Change in prevalence, 1999-2018	+3.60 (+1.11 to +6.09)	.01	+4.49 (+3.12 to +5.86)	< .001	+4.35 (+2.84 to +5.85)	< .001	+4.07 (+3.46 to +4.68)	< .001
Prevalence in 1999	3.78 (2.60 to 5.47)	NA	2.90 (2.34 to 3.57)	NA	4.06 (3.40 to 4.84)	NA	3.78 (3.50 to 4.08)	NA
Prevalence in 2018	7.38 (5.61 to 9.66)	NA	7.39 (6.28 to 8.69)	NA	8.41 (7.17 to 9.83)	NA	7.85 (7.32 to 8.40)	NA
Change in difference with White individuals, 1999-2018	−0.47 (−3.03 to +2.09)	.72	+0.42 (−1.08 to +1.92)	.58	+0.27 (−1.35 to +1.90)	.74	NA	NA
Difference in 1999	0.00 (−1.45 to +1.45)	.99	−0.88 (−1.56 to −0.20)	.01	+0.28 (−0.49 to +1.05)	.47	NA	NA
Difference in 2018	−0.47 (−2.58 to +1.64)	.66	−0.46 (−1.80 to +0.88)	.50	+0.55 (−0.88 to +1.99)	.45	NA	NA
Long waiting times at office/clinic								
Change in prevalence, 1999-2018	+2.60 (+0.98 to +4.22)	.002	+2.73 (+1.43 to +4.02)	< .001	+3.86 (+2.42 to +5.29)	< .001	+1.23 (+0.80 to +1.66)	< .001
Prevalence in 1999	1.87 (1.11 to 3.15)	NA	3.08 (2.50 to 3.79)	NA	3.80 (3.17 to 4.55)	NA	2.45 (2.23 to 2.71)	NA
Prevalence in 2018	4.47 (3.40 to 5.87)	NA	5.81 (4.81 to 7.02)	NA	7.65 (6.49 to 8.99)	NA	3.68 (3.34 to 4.05)	NA
Change in difference with White individuals, 1999-2018	+1.37 (−0.31 to +3.05)	.11	+1.50 (+0.13 to +2.86)	.03	+2.63 (+1.13 to +4.12)	< .001	NA	NA
Difference in 1999	−0.59 (−1.64 to +0.47)	.27	+0.63 (−0.07 to +1.32)	.08	+1.34 (+0.61 to +2.08)	< .001	NA	NA
Difference in 2018	+0.78 (−0.52 to +2.09)	.24	+2.12 (+0.95 to +3.30)	< .001	+3.97 (+2.66 to +5.27)	< .001	NA	NA
Inconvenient office hours								
Change in prevalence from 1999-2018	+1.28 (−0.48 to +3.03)	.15	+1.45 (+0.46 to +2.43)	.004	+1.81 (+0.79 to +2.83)	< .001	+1.58 (+1.14 to +2.03)	< .001
Prevalence in 1999	2.14 (1.28 to 3.64)	NA	1.71 (1.33 to 2.19)	NA	2.04 (1.61 to 2.58)	NA	2.23 (2.01 to 2.47)	NA
Prevalence in 2018	3.41 (2.37 to 4.94)	NA	3.15 (2.40 to 4.14)	NA	3.85 (3.05 to 4.85)	NA	3.81 (3.45 to 4.20)	NA
Change in difference with White individuals, 1999-2018	−0.31 (−2.12 to +1.50)	.74	−0.14 (−1.22 to +0.94)	.80	+0.22 (−0.89 to +1.34)	.69	NA	NA
Difference in 1999	−0.09 (−1.30 to +1.12)	.89	−0.52 (−1.01 to −0.03)	.04	−0.19 (−0.72 to +0.35)	.50	NA	NA
Difference in 2018	−0.40 (−1.74 to +0.95)	.57	−0.66 (−1.62 to +0.30)	.18	+0.04 (−0.93 to +1.01)	.94	NA	NA
Lack of transportation								
Change in prevalence from 1999-2018	+0.38 (−1.24 to +2.00)	.65	+2.73 (+1.48 to +3.99)	< .001	+1.01 (+0.24 to +1.78)	.01	+0.92 (+0.64 to +1.19)	< .001
Prevalence in 1999	1.70 (0.88 to 3.21)	NA	2.35 (1.84 to 3.02)	NA	1.71 (1.32 to 2.21)	NA	0.79 (0.68 to 0.92)	NA
Prevalence in 2018	2.07 (1.25 to 3.43)	NA	5.09 (4.11 to 6.32)	NA	2.71 (2.15 to 3.41)	NA	1.71 (1.48 to 1.97)	NA
Change in difference with White individuals, 1999-2018	−0.54 (−2.18 to +1.10)	.52	+1.82 (+0.53 to +3.10)	.01	+0.09 (−0.73 to +1.38)	.83	NA	NA
Difference in 1999	+0.90 (−0.29 to +2.10)	.14	+1.56 (+0.96 to +2.16)	< .001	+0.91 (+0.45 to +1.38)	< .001	NA	NA
Difference in 2018	+0.36 (−0.76 to +1.49)	.53	+3.38 (+2.24 to +4.51)	< .001	+1.00 (+0.33 to +1.68)	.004	NA	NA

^a^ For change in prevalence and change in difference, a positive sign (+) means the prevalence of each indicator (or its difference with White respondents) increased and a negative sign (−) means it decreased. Estimates were adjusted by age and US region. Income- and sex-stratified estimates are available in eTable 4 in the Supplement.

When estimates were stratified by sex, the prevalence of these barriers to timely medical care increased over time among both males and females, although the prevalence was comparatively higher among females than among males. The racial and ethnic gap increased only among males (eTable 4 in the Supplement). In 2018, compared with White males, the estimated prevalence was 3.2 pp higher among Black males (95% CI, 0.4-6.0; *P* = .03) and 3.7 pp higher among Hispanic/Latino males (95% CI, 1.0-6.4; *P* = .01).

When analyzed by income level, there were no significant changes in the differences between subgroups during the study period (eTable 4 in the Supplement), and the 1999 and 2018 differences between White and Black individuals were not significant within each income stratum. Among those with middle to high income, the prevalence of barriers to timely medical care was 3.5 pp higher among Hispanic/Latino respondents compared with non-Hispanic White respondents (95% CI, 1.1-6.0; *P* = .01).

In a sensitivity analysis, we stratified the analysis by insurance status and presence of barriers related to the affordability of care (eTable 5 and eFigures 2 and 3 in the Supplement). When compared with uninsured White individuals, disparities in any barrier to timely medical care increased from 1999 to 2018 among uninsured Black and Hispanic/Latino individuals (+6.6 pp and +5.3 pp, respectively; *P* = .03 each; as shown in eTable 3 in the Supplement), reaching a 2018 difference of +7.2 pp (95% CI, 1.9-12.5; *P* = .01) and +4.5 pp (95% CI, 0.5-8.6; *P* = .03), respectively. Of note, insured Hispanic/Latino respondents also had higher prevalence of these barriers compared with White respondents in 2018 (+3.5 pp; 95% CI, 1.5-5.6; *P* < .001). There were no significant differences between insured Black respondents and White respondents during the study period. In a separate analysis, the disparities in barriers not directly related to cost of care between White individuals and Black individuals increased among those who also experienced barriers related to affordability of care, reaching +6.7 pp in 2018 (95% CI, 1.1-12.4; *P* = .02), but not among those who did not experience affordability barriers. Hispanic/Latino respondents reported a higher prevalence of these barriers, regardless of whether they also experienced affordability barriers or not, as shown in eFigure 3 and eTable 5 in the Supplement.

#### Specific Barriers

**Figure 3.**
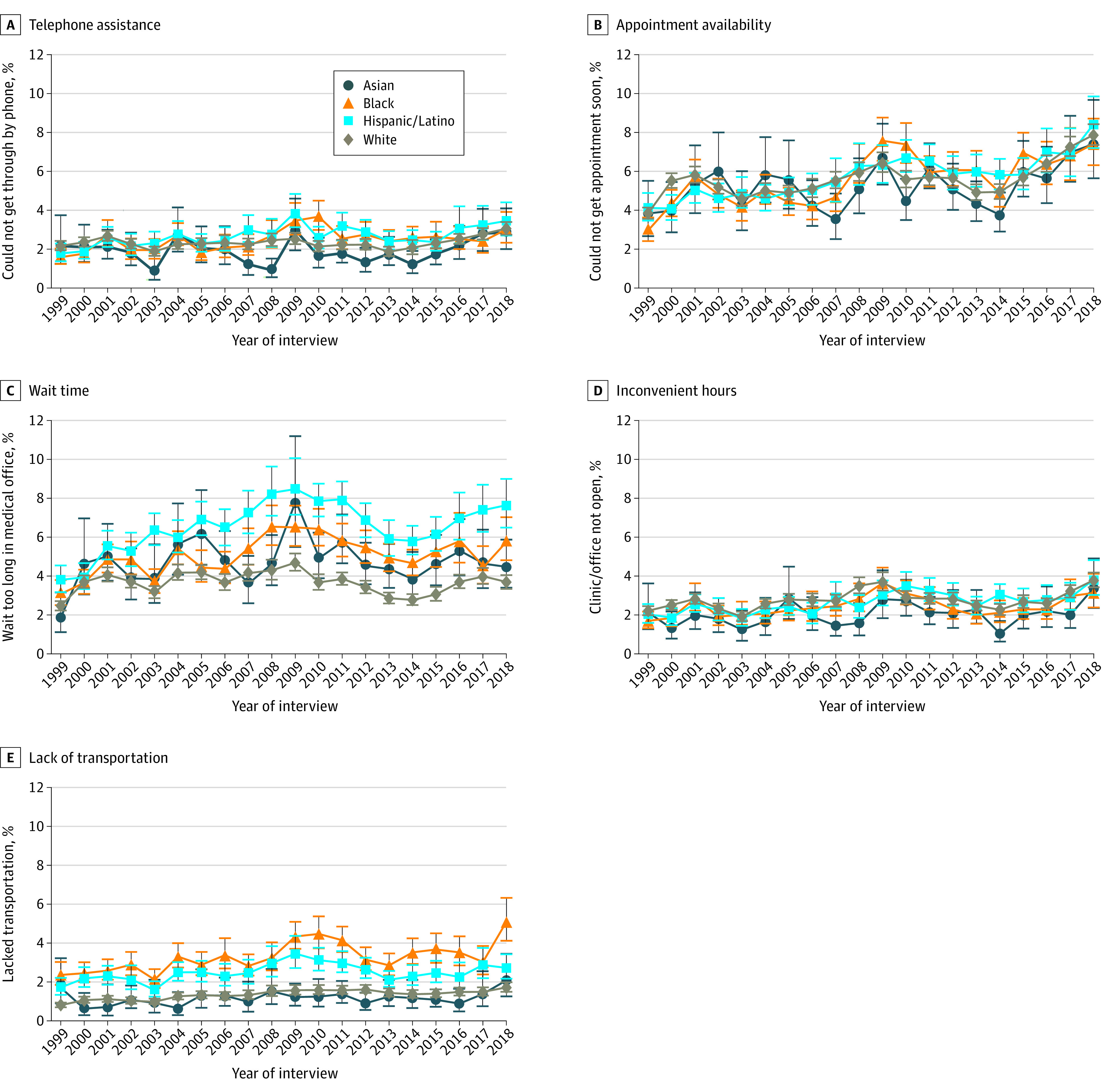
Trends in Annual Prevalence of Specific Barriers to Timely Medical Care Not Directly Related to Cost of Care Among US Adults, by Race and Ethnicity, National Health Interview Survey, 1999 to 2018 Brackets represent 95% CIs. All estimates were adjusted by age and US region.

During the study period, each of the 5 barriers significantly increased in prevalence among Black, Hispanic/Latino, and White respondents (Figure 3, Table 2; eTable 6 in the Supplement). Among Asian respondents, the increase occurred only in the proportion of those who reported having delayed care because of long waiting times and because they could not get an appointment soon enough (+2.6 [95% CI, 1.0-4.2; *P* = .002] and +3.6 [95% CI, 1.1-6.1 pp; *P* = .01], respectively).

There was a significant increase in the difference in prevalence between Black individuals and White individuals who reported delaying care because of long waiting times at the medical office or a lack of transportation (increased by 1.5 pp [95% CI, 0.1-2.9; *P* = .03] and 1.8 pp [95% CI, 0.5-3.1; *P* = .01], respectively). In 2018, compared with the prevalence of each of these 2 barriers among White respondents (3.7% [95% CI, 3.3%- 4.1%] and 1.7% [95% CI, 1.5%-2.0%], respectively), the prevalence rates were higher by 2.1 pp; (95% CI, 1.0-3.3) and 3.4 pp (95% CI, 2.2-4.5) among Black individuals, respectively (*P* < .01 for each). Such differences were still significant when stratified by sex and income level (eTables 9 and 11 in the Supplement). However, among those with middle to high income, the difference between Black respondents and White respondents who reported delaying care because of long waiting times was not significant in 2018 (eTable 9 in the Supplement).

In addition, the difference in prevalence between Hispanic/Latino and White respondents who reported delaying care because of long waiting times widened significantly, increasing by 2.6 pp (95% CI, 1.1-4.1; *P* < .001). In 2018, compared with the prevalence among White individuals, the proportion of Hispanic/Latino individuals who experienced this barrier was 4.0 pp higher (95% CI, 2.7-5.3; *P* < .001). This difference was still significant when stratified by sex and income level (eTable 6 in the Supplement). In the same year, the prevalence of Hispanic/Latino respondents who reported delayed care because of lack of transportation was 1.0 pp higher (95% CI, 0.3, 1.7; *P* = .004; Table 2) than that of White respondents, with the difference mainly among females and individuals of middle or high income (eTable 11 in the Supplement).

The change in prevalence difference between subgroups for the other 3 barriers from 1999–2018 was not significant (Table 2), with little variation by sex or household income level (eFigures 4-8 and eTables 7-11 in the Supplement).

## Discussion

In this nationally representative study, we found that from 1999 to 2018, the overall estimated proportion of respondents who reported barriers to timely care nearly doubled, increasing from 7.1% to 13.5%, and the increase was not proportionate across the 4 race and ethnicity groups. During this period, differences in accessibility and availability of care between White respondents and Black and Latino respondents increased. In 2018, Black and Latino respondents were more likely to report delayed care because of lack of transportation and long waiting times at the doctor’s office compared with White respondents.

This study expands the evidence in several ways. First, to the best of our knowledge, this is the first investigation to show worsening racial and ethnic disparities in barriers to timely medical care not directly related to cost of care over 20 years. Several studies have reported disparities in some of these measures,^9,10,25^ even in recent years^8^; however, this study distinguishes itself by quantifying how these disparities have changed during an extended period. Second, our evaluation of trends among race and ethnicity groups regarding 5 specific barriers to timely medical care provides a more comprehensive picture than previous studies, some of which found increasing trends in some of these indicators, but not in all 5, and did not describe trends by race and ethnicity.^12,17^ We found that the overall prevalence of these barriers increased during the 20-year period, but at disparate rates across the 4 race and ethnicity groups studied. Third, we described how the increases in disparities in access to timely medical care occurred mostly among men and were attenuated when stratified by income level. This study is the first, to our knowledge, to evaluate how these racial and ethnic disparities changed by sex and income level.

These findings have several important health policy implications. First, the increase in prevalence in barriers across race and ethnicity groups in the US indicates a worsening societal failure to deliver timely medical care. The fact that, overall, nearly 1 in 7 adults in 2018 experienced barriers to timely medical care indicates that attempts to improve access to care through improving access to insurance coverage alone may be inadequate—and may not be enough to reduce disparities. We found that there was no significant trend in the racial and ethnic disparities after 2010 (when the ACA was signed into law). Moreover, the sensitivity analysis showed that the disparities in these barriers increased mainly among those who were uninsured; however, significant differences in prevalence rates also existed between Hispanic/Latino individuals and White individuals—even among the insured. Although increasing insurance coverage may address unmet medical needs by reducing cost, it is less clear that it removes barriers to timely medical care that are not directly related to cost. In the years after the ACA Medicaid expansions, a study found increases in delayed care because of long waiting times and inability to schedule an appointment soon enough, particularly among those with low income.^26^ Similarly, there was no difference in the prevalence of these 2 barriers (long waiting times and appointment availability) by Medicare eligibility status (ie, those <65 years vs ≥65 years old).^27^ These observations—along with our finding that racial and ethnic disparities remained stable in the years after the ACA—underscore the need for renewed national investments in measuring, tracking, and improving primary care availability and accessibility per the broader social determinants of health.

Second, the growing racial and ethnic disparities in prevalence of these barriers to timely medical care suggest that the scope of national efforts to eliminate disparities in health care access should be expanded and include societal reforms beyond the health care system. This is not to say that health care−specific interventions (eg, the ACA, the national Culturally and Linguistically Appropriate Services) are not fundamental toward this goal, but that eliminating disparities in these indicators requires that policy interventions address nonmedical barriers to health care access and quality, including education, housing, urban planning, employment, and transportation, which disproportionately affect underserved populations.^28^ These interventions should be implemented in the context of structural racism that accentuates barriers to accessing medical care for minority groups, both within and adjacent to the health care system. Importantly, there is evidence that because of historical segregation of racial and ethnic minority groups, Black and Latino individuals are more likely to live in medically underserved areas, to receive worse quality of care, and to visit the emergency department for primary care−treatable conditions.^29,30,31^ These disparities are further compounded by transportation barriers.^17^ Thus, there is a need for a multisectoral effort to improve spatial accessibility to high-quality primary care clinics and health care professionals for minoritized race and ethnicity groups. Strategies could include addressing differences in distribution of health care facilities, increasing flexibility of care (eg, implementing urgent clinics that do not result in discontinuity of care), including insurance coverage for nonemergency transportation to medical care, and leveraging digital health technologies for high-quality telehealth consultations that are available and accessible.

Third, there are important implications from the income- and sex-stratified findings. The finding that racial and ethnic disparities were attenuated by lower income serves as an example of the pervasiveness of income inequality in access to health care, even beyond cost-related indicators. Regarding sex, although racial and ethnic disparities among women were mostly static, they had an overall higher prevalence of barriers during the study period compared with men of the same race or ethnicity. Because women face structural challenges to accessing sex-specific primary care (eg, pregnancy, menopause, gender-sensitive care^32^), these findings add to the evidence of a need to improve women’s access to primary care throughout the different stages of the life cycle.

### Limitations

This study had some limitations. First, we measured some but not all important barriers that are not directly related to cost of care (eg, language barriers, technology access). Second, NHIS data are self-reported, and the data lacked information on what type of care was delayed by the measured barriers. Third, there is no information regarding state or rural or urban setting of residence, which may have influenced some of the measures. However, these limitations did not affect the primary findings regarding self-reported barriers during the past 2 decades.

## Conclusions

The findings of this serial cross-sectional study of NHIS data suggest that from 1999 to 2018, barriers to timely medical care increased for all 4 race and ethnicity groups studied. Moreover, there were increasing differences between groups for some of the barriers. Compared with White individuals, Black and Hispanic/Latino individuals were more likely to report experiencing these barriers. There is considerable scope for implementing changes to remove the barriers to medical care and to eliminate these racial and ethnic disparities.

## Notes

Supplement.
eMethods.
**eFigure 1.** Study Population Flowchart **eFigure 2.** Sensitivity Analysis: Trends in Annual Prevalence of Barriers to Timely Medical Care Among US Adults by Race and Ethnicity, Stratified by Insurance Status **eFigure 3.** Sensitivity Analysis: Trends in Annual Prevalence of Barriers to Timely Medical Care Among US Adults by Race and Ethnicity, Stratified by Presence of Affordability Barriers to Care **eFigure 4.** Trends in Proportion of Individuals Reporting Delaying Care Because They Couldn’t Get Through by Phone by Race and Ethnicity, Stratified by Sex and Income Level **eFigure 5.** Trends in Proportion of Individuals Reporting Delaying Care Because They Couldn’t Get an Appointment Soon Enough by Race and Ethnicity, Stratified by Sex and Income Level **eFigure 6.** Trends in Proportion of Individuals Reporting Delaying Care Because They Had to Wait Too Long to See the Doctor by Race and Ethnicity, Stratified by Sex and Income Level **eFigure 7.** Trends in Proportion of Individuals Reporting Delaying Care Because the Doctor’s Office Was Not Open When They Could Get There by Race and Ethnicity, Stratified by Sex and Income Level **eFigure 8.** Trends in Proportion of Individuals Reporting Delaying Care Because They Lacked Transportation by Race and Ethnicity, Stratified by Sex and Income Level **eTable 1.** Study Population Characteristics by Years **eTable 2.** Annualized Rate of Change in Prevalence of and Racial Differences in Barriers to Timely Medical Care **eTable 3.** Annual Prevalence of Ordered Number of Barriers by Race and Ethnicity **eTable 4.** Change in the Adjusted Prevalence of Any Barriers to Timely Medical Care from 1999 to 2018, by Race and Ethnicity and Stratified by Sex and Income Level **eTable 5.** Sensitivity Analysis: Change in the Adjusted Prevalence of Any Barriers to Timely Medical Care Access from 1999 to 2018, by Race and Ethnicity and Stratified by Insurance Status and Presence of Affordability Barriers to Care **eTable 6.** Annual Estimates (95% CI) of Each Barrier to Timely Medical Care by Race and Ethnicity **eTable 7.** Change in the Adjusted Proportion of Individuals Reporting Delaying Care Because They Couldn’t Get Through by Phone from 1999 to 2018, by Race and Ethnicity and Stratified by Sex and Income Level **eTable 8.** Change in the Adjusted Proportion of Individuals Reporting Delaying Care Because They Couldn’t Get an Appointment Soon Enough from 1999 to 2018, by Race and Ethnicity and Stratified by Sex and Income Level **eTable 9.** Change in the Adjusted Proportion of Individuals Reporting Delaying Care Because They Had to Wait Too Long to See the Doctor from 1999 to 2018, by Race and Ethnicity and Stratified by Sex and Income Level **eTable 10.** Change in the Adjusted Proportion of Individuals Reporting Delaying Care Because the Doctor’s Office Was Not Open When They Could Get There from 1999 to 2018, by Race and Ethnicity and Stratified by Sex and Income Level **eTable 11.** Change in the Adjusted Proportion of Individuals Reporting Delaying Care Because They Lacked Transportation from 1999 to 2018, by Race and Ethnicity and Stratified by Sex and Income Level
