# Supplementary material for: Trends in Racial and Ethnic Disparities in Barriers to Timely Medical Care Among Adults in the US, 1999 to 2018
Source: JAMA Health Forum. 2022 Oct 28;3(10):e223856. doi: 10.1001/jamahealthforum.2022.3856 (PMC9617175; doi:10.1001/jamahealthforum.2022.3856)
Supplement: Supplement. — eMethods. eFigure 1. Study Population Flowchart eFigure 2. Sensitivity Analysis: Trends in Annual Prevalence of Barriers to Timely Medical Care Among US Adults by Race and Ethnicity, Stratified by Insurance Status eFigure 3. Sensitivity Analysis: Trends in Annual Prevalence of Barriers to Timely Medical Care Among US Adults by Race and Ethnicity, Stratified by Presence of Affordability Barriers to Care eFigure 4. Trends in Proportion of Individuals Reporting Delaying Care Because They Couldn’t Get Through by Phone by Race and Ethnicity, Stratified by Sex and Income Level eFigure 5. Trends in Proportion of Individuals Reporting Delaying Care Because They Couldn’t Get an Appointment Soon Enough by Race and Ethnicity, Stratified by Sex and Income Level eFigure 6. Trends in Proportion of Individuals Reporting Delaying Care Because They Had to Wait Too Long to See the Doctor by Race and Ethnicity, Stratified by Sex and Income Level eFigure 7. Trends in Proportion of Individuals Reporting Delaying Care Because the Doctor’s Office Was Not Open When They Could Get There by Race and Ethnicity, Stratified by Sex and Income Level eFigure 8. Trends in Proportion of Individuals Reporting Delaying Care Because They Lacked Transportation by Race and Ethnicity, Stratified by Sex and Income Level eTable 1. Study Population Characteristics by Years eTable 2. Annualized Rate of Change in Prevalence of and Racial Differences in Barriers to Timely Medical Care eTable 3. Annual Prevalence of Ordered Number of Barriers by Race and Ethnicity eTable 4. Change in the Adjusted Prevalence of Any Barriers to Timely Medical Care from 1999 to 2018, by Race and Ethnicity and Stratified by Sex and Income Level eTable 5. Sensitivity Analysis: Change in the Adjusted Prevalence of Any Barriers to Timely Medical Care Access from 1999 to 2018, by Race and Ethnicity and Stratified by Insurance Status and Presence of Affordability Barriers to Care eTable 6. Annual Estimates (95% CI) of Each Barrier to Tim [file jamahealthforum-e223856-s001.pdf]

Supplemental Online Content

Caraballo C, Ndumele CD, Roy B, et al. Trends in racial and ethnic disparities in barriers to timely medical care among adults in the US, 1999 to 2018. *JAMA Health Forum.* 2022;3(10):e223856. doi:10.1001/jamahealthforum.2022.3856

eMethods.

eFigure 1. Study Population Flowchart

eFigure 2. Sensitivity Analysis: Trends in Annual Prevalence of Barriers to Timely Medical Care Among US Adults by Race and Ethnicity, Stratified by Insurance Status

eFigure 3. Sensitivity Analysis: Trends in Annual Prevalence of Barriers to Timely Medical Care Among US Adults by Race and Ethnicity, Stratified by Presence of Affordability Barriers to Care

eFigure 4. Trends in Proportion of Individuals Reporting Delaying Care Because They Couldn't Get Through by Phone by Race and Ethnicity, Stratified by Sex and Income Level

eFigure 5. Trends in Proportion of Individuals Reporting Delaying Care Because They Couldn't Get an Appointment Soon Enough by Race and Ethnicity, Stratified by Sex and Income Level

eFigure 6. Trends in Proportion of Individuals Reporting Delaying Care Because They Had to Wait Too Long to See the Doctor by Race and Ethnicity, Stratified by Sex and Income Level

eFigure 7. Trends in Proportion of Individuals Reporting Delaying Care Because the Doctor's Office Was Not Open When They Could Get There by Race and Ethnicity, Stratified by Sex and Income Level

eFigure 8. Trends in Proportion of Individuals Reporting Delaying Care Because They Lacked Transportation by Race and Ethnicity, Stratified by Sex and Income Level

eTable 1. Study Population Characteristics by Years

eTable 2. Annualized Rate of Change in Prevalence of and Racial Differences in Barriers to Timely Medical Care

eTable 3. Annual Prevalence of Ordered Number of Barriers by Race and Ethnicity

© 2022 Caraballo C et al. *JAMA Health Forum.*

eTable 4. Change in the Adjusted Prevalence of Any Barriers to Timely Medical Care from 1999 to 2018, by Race and Ethnicity and Stratified by Sex and Income Level

eTable 5. Sensitivity Analysis: Change in the Adjusted Prevalence of Any Barriers to Timely Medical Care Access from 1999 to 2018, by Race and Ethnicity and Stratified by Insurance Status and Presence of Affordability Barriers to Care

eTable 6. Annual Estimates (95% CI) of Each Barrier to Timely Medical Care by Race and Ethnicity

eTable 7. Change in the Adjusted Proportion of Individuals Reporting Delaying Care Because They Couldn't Get Through by Phone from 1999 to 2018, by Race and Ethnicity and Stratified by Sex and Income Level

eTable 8. Change in the Adjusted Proportion of Individuals Reporting Delaying Care Because They Couldn't Get an Appointment Soon Enough from 1999 to 2018, by Race and Ethnicity and Stratified by Sex and Income Level

eTable 9. Change in the Adjusted Proportion of Individuals Reporting Delaying Care Because They Had to Wait Too Long to See the Doctor from 1999 to 2018, by Race and Ethnicity and Stratified by Sex and Income Level

eTable 10. Change in the Adjusted Proportion of Individuals Reporting Delaying Care Because the Doctor's Office Was Not Open When They Could Get There from 1999 to 2018, by Race and Ethnicity and Stratified by Sex and Income Level

eTable 11. Change in the Adjusted Proportion of Individuals Reporting Delaying Care Because They Lacked Transportation from 1999 to 2018, by Race and Ethnicity and Stratified by Sex and Income Level

This supplemental material has been provided by the authors to give readers additional information about their work.

© 2022 Caraballo C et al. *JAMA Health Forum.*

eMethods

About the National Health Interview Survey

The National Health Interview Survey (NHIS) consists of a questionnaire divided into 4 cores: Household Composition, Family Core, Sample Child Core, and Sample Adult Core. The Household Composition file collects basic and relationship information about all persons in a household. The Family Core file collects sociodemographic characteristics, basic indicators of health status, activity limitations, injuries, health insurance coverage, and access to and utilization of health care services. From each family, one sample child and one sample adult are randomly selected to gather more in-depth information for the Sample Child Core and Sample Adult Core, respectively.[1]

Demographic Variables: Race and Ethnicity

Persons that identified themselves as Hispanic/Latino were grouped as Hispanic/Latino, regardless of their race selection. Those that selected a race and did not identify themselves as Hispanic/Latino, were grouped based on their primary race selection.[2,3] Due to small numbers of participants, we excluded non-Hispanic individuals who identified as "other" race, did not identify with a primary race, or identified as Alaskan Native or non-Hispanic American Indian. Of note, for confidentiality reasons, in the publicly available NHIS data, individuals that self-reported their racial background as Native Hawaiian or Other Pacific Islander were included in the "other race" category from 1999-2002, and did not have their race group released from 2003-2018.[1,4]

Statistical Analysis

In the multivariable logistic regressions to estimate the annual prevalence of each barrier to timely medical care by race and ethnicity, the barrier was the dependent variable, and age and region were the independent variables. Each year's coefficient—combined with the intercept—represented the logit of the adjusted annual proportion of each outcome. Then, we used the results to create estimated annual prevalence by using the inverse logit of each year effect as the annual prevalence, and applying the method of parametric bootstrapping to calculate the standard error (SE) and the confidence interval (CI) for the transformed coefficients.[5]

To estimate the annual low-income prevalence by race and ethnicity, we used the mean annual estimate obtained by separate logistic regressions using a similar approach described in the main analysis, but with each of the multiply imputed low-income variables as the dependent variable and an indicator for each year as the independent variables. Similarly, we used the mean prevalence estimate of each barrier to timely medical care indicator from separate regressions for each of the income groups (low-income and middle/high-income).[6]

In the ordered logistic regression models to estimate the proportion of individuals with 0, 1, 2, 3, or ≥4 specific barriers over the years, the number of barriers was the dependent variable and age, region, and an indicator for each year were the independent variables, obtaining the estimates for each race and ethnicity group separately.

Disparities in health care are often driven by underlying disparities in comorbidities, employment, education, and social support. Because our goal was to measure total disparities, we did not adjust for any of these characteristics in the model, since doing so would likely obscure the differences we were trying to measure.

For the trend analysis, rather than assuming a monotonic relationship between time and prevalence, we graphically assessed the relationship of prevalence of the indicator for any barrier to timely medical care. Based on this assessment, we modeled time as a linear spline with knots at 2010 and 2014 to reflect the observed inflection points. We then used the coefficient of the time variables to evaluate the slope of each outcome's prevalence during each period.

References

1. National Center for Health Statistics. About the National Health Interview Survey. 2020 (https://www.cdc.gov/nchs/nhis/about_nhis.htm).

© 2022 Caraballo C et al. *JAMA Health Forum.*

2. Hoopes MJ, Angier H, Gold R, et al. Utilization of community health centers in Medicaid expansion and nonexpansion states, 2013-2014. J Ambul Care Manage 2016;39(4):290-8. (In eng). DOI: 10.1097/jac.0000000000000123.
3. Yoon P, Hall J, Fuld J, et al. Alternative methods for grouping race and ethnicity to monitor COVID-19 outcomes and vaccination coverage. Morbidity and Mortality Weekly Report 2021;70(32):1075.
4. Centers for Disease Control and Prevention. National Health Interview Survey, 1997-2018. Survey description document. (https://www.cdc.gov/nchs/nhis/1997-2018.htm).
5. King G, Tomz M, Wittenberg J. Making the most of statistical analyses: Improving interpretation and presentation. Am J Pol Sci 2000:347-361.
6. Division of Health Interview Statistics National Center for Health Statistics. Multiple imputation of family income and personal earnings in the National Health Interview Survey: methods and examples. August 2019. (https://nhis.ipums.org/nhis/resources/tecdoc18.pdf).

eFigure 1. Study Population Flowchart

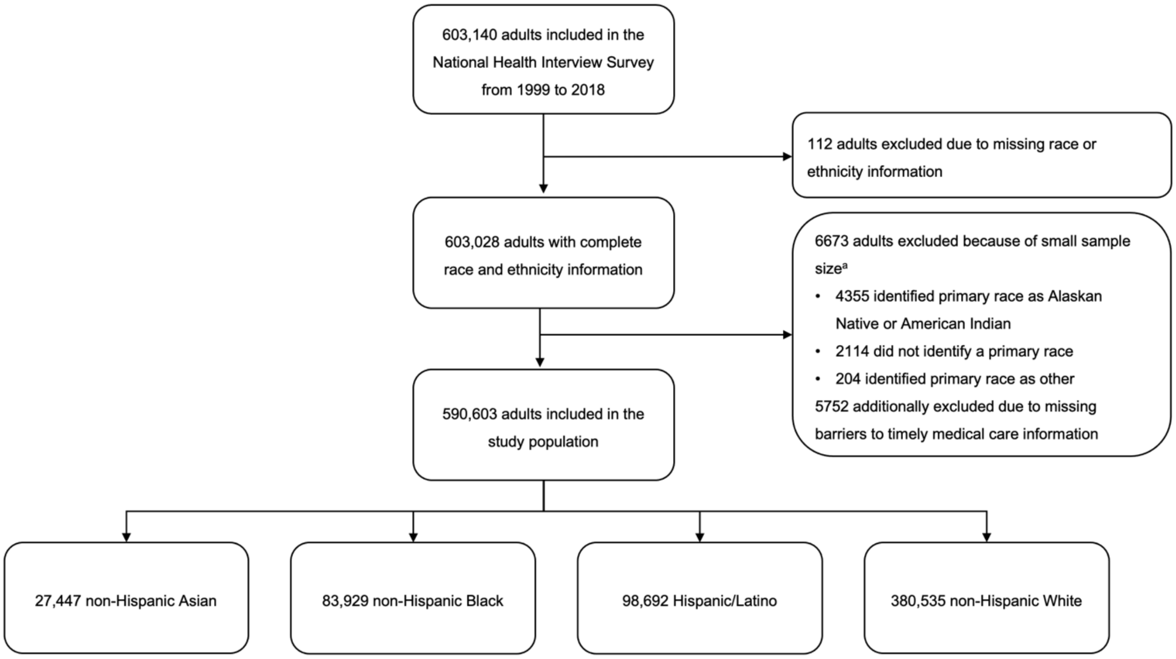

The 4 mutually exclusive racial/ethnic subgroups were created based on the primary race and ethnicity combination.
[a] These excluded individuals also did not identify as Hispanic/Latino.

© 2022 Caraballo C et al. *JAMA Health Forum.*

eFigure 2. Sensitivity Analysis: Trends in Annual Prevalence of Barriers to Timely Medical Care Among US Adults by Race and Ethnicity, Stratified by Insurance Status

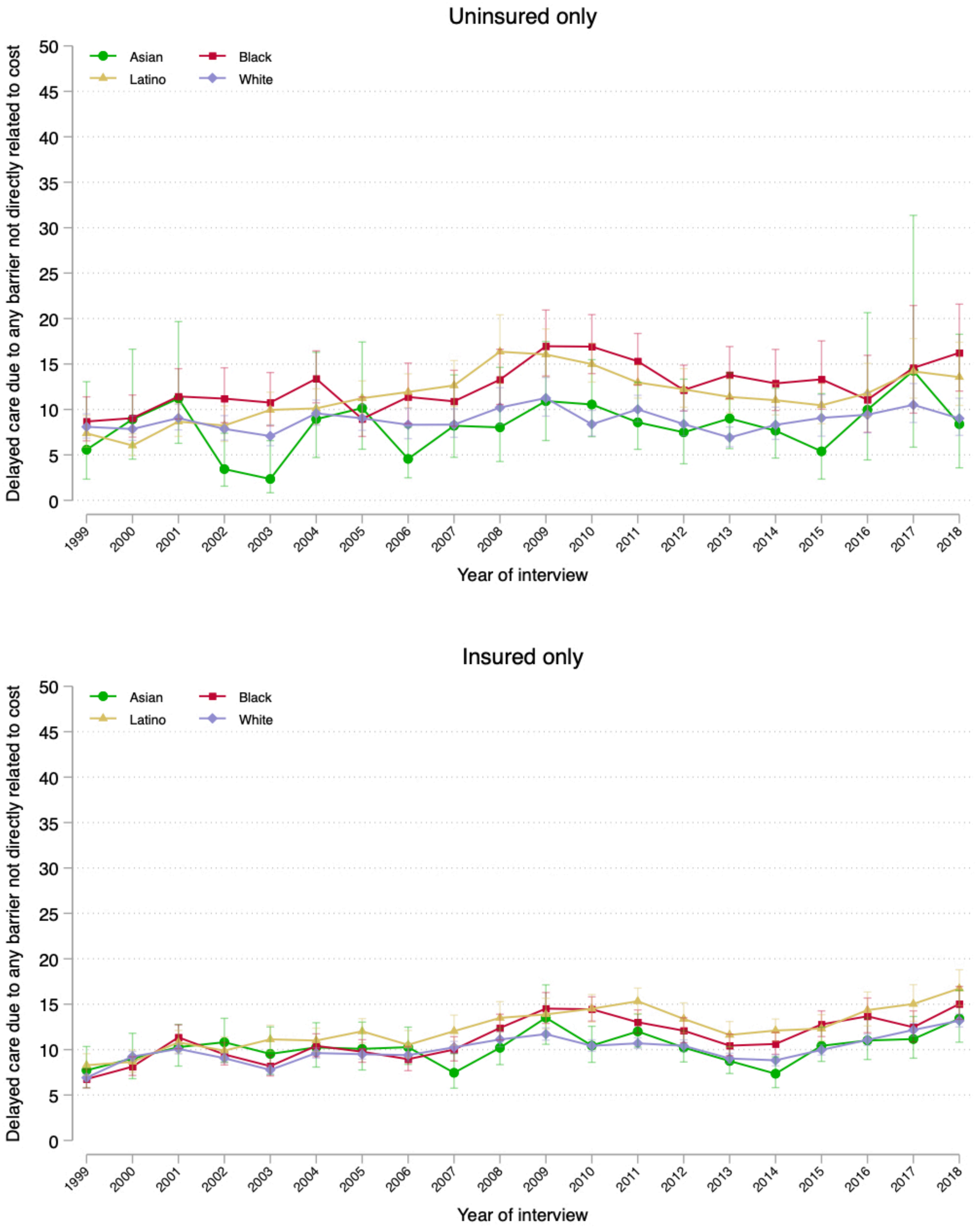

© 2022 Caraballo C et al. *JAMA Health Forum.*

eFigure 3. Sensitivity Analysis: Trends in Annual Prevalence of Barriers to Timely Medical Care Among US Adults by Race and Ethnicity, Stratified by Presence of Affordability Barriers to Care

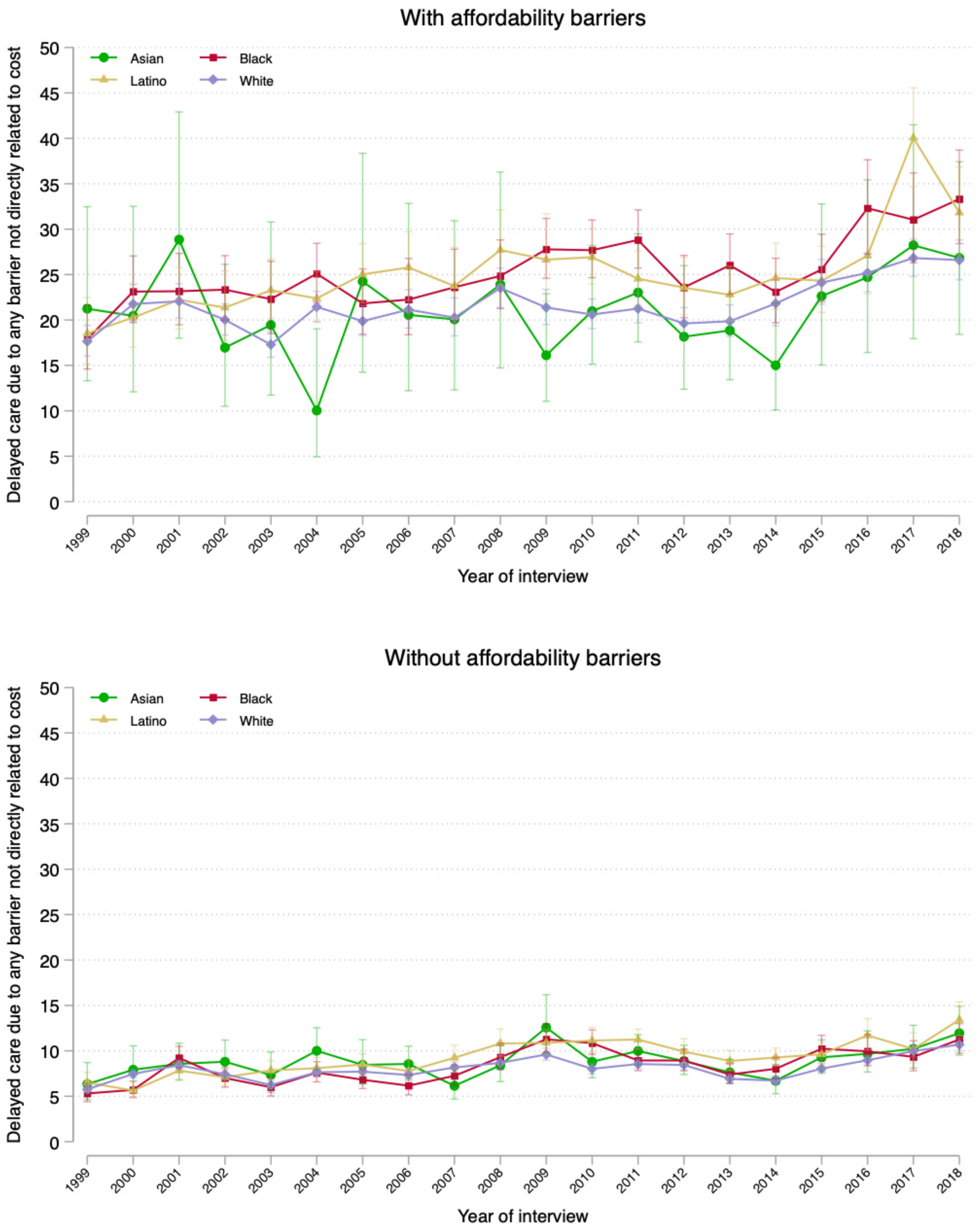

© 2022 Caraballo C et al. *JAMA Health Forum.*

eFigure 4. Trends in Proportion of Individuals Reporting Delaying Care Because They Couldn't Get Through by Phone by Race and Ethnicity, Stratified by Sex and Income Level

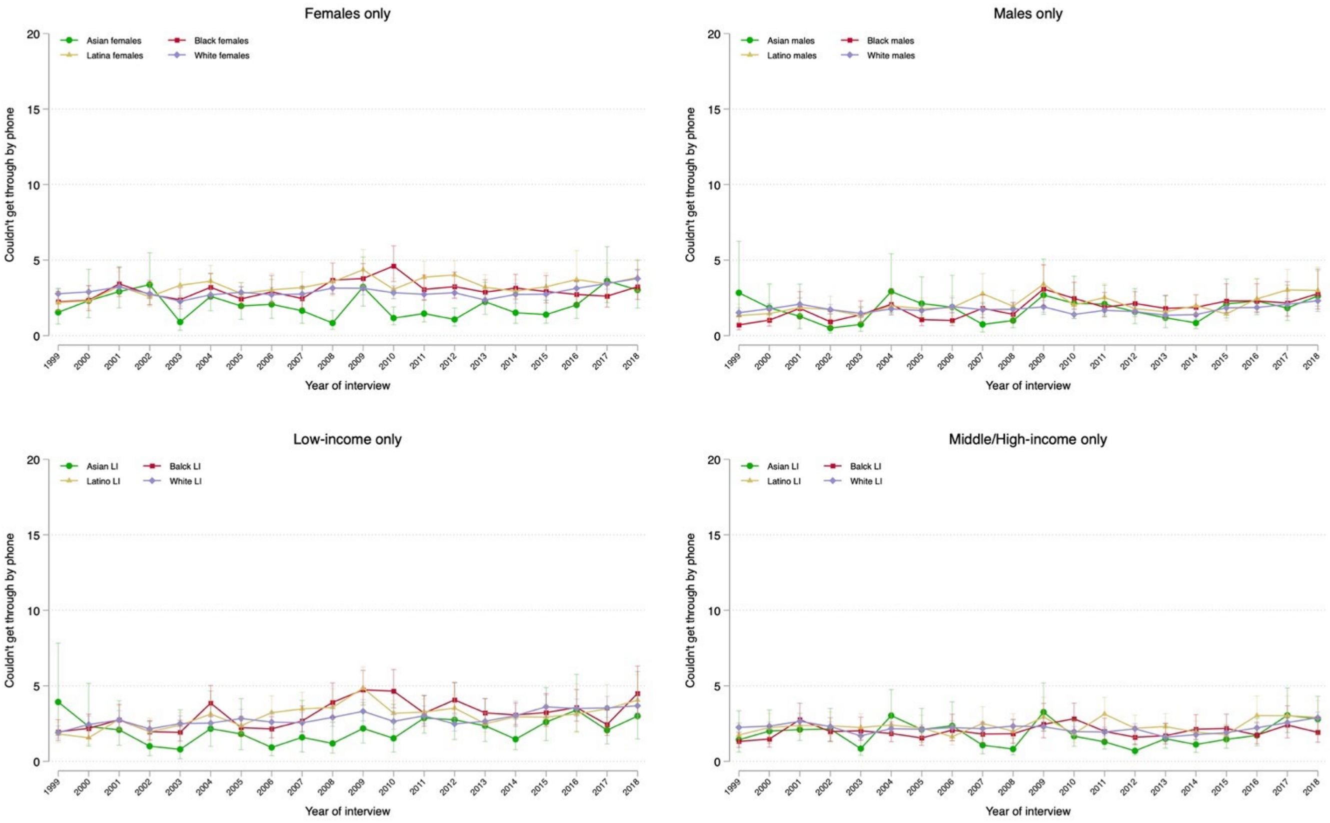

© 2022 Caraballo C et al. *JAMA Health Forum.*

eFigure 5. Trends in Proportion of Individuals Reporting Delaying Care Because They Couldn't Get an Appointment Soon Enough by Race and Ethnicity, Stratified by Sex and Income Level

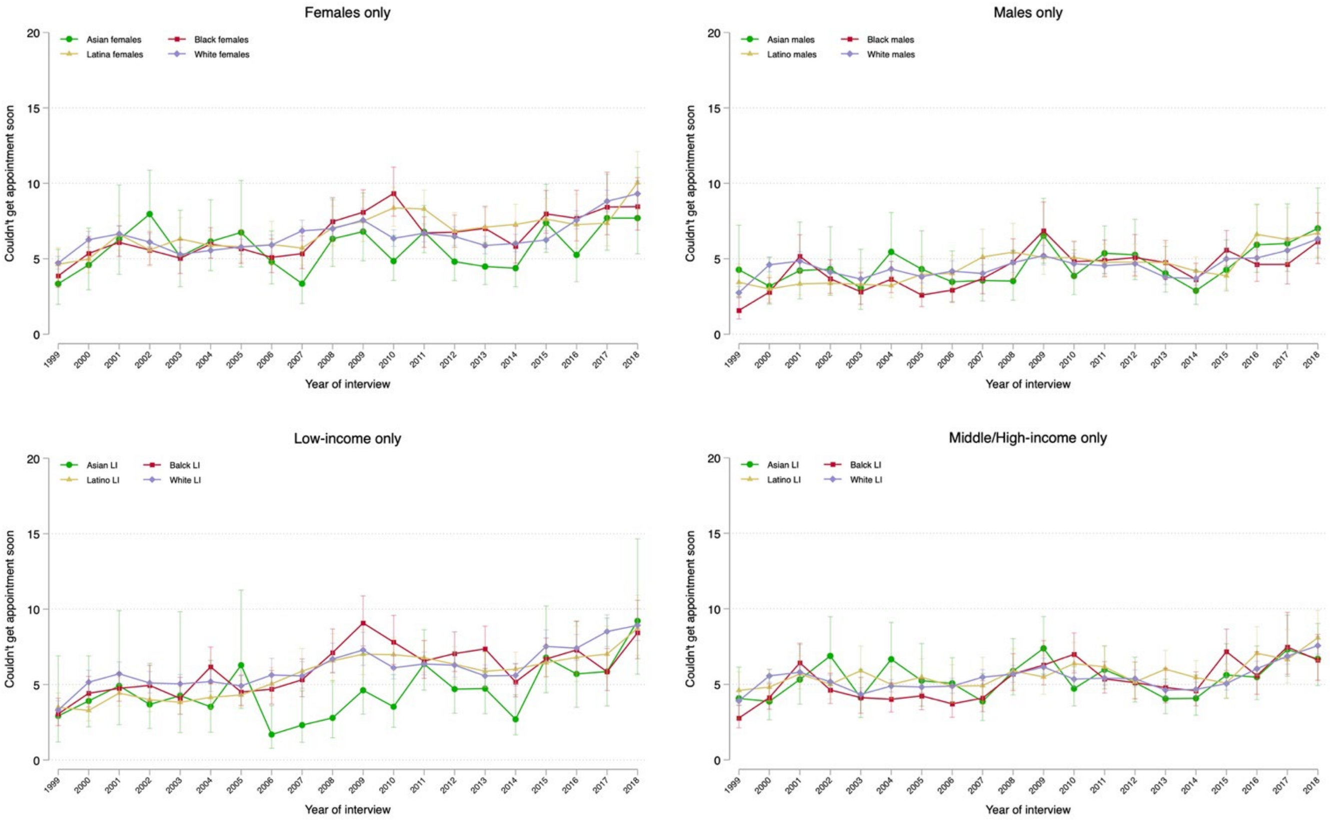

© 2022 Caraballo C et al. *JAMA Health Forum.*

eFigure 6. Trends in Proportion of Individuals Reporting Delaying Care Because They Had to Wait Too Long to See the Doctor by Race and Ethnicity, Stratified by Sex and Income Level

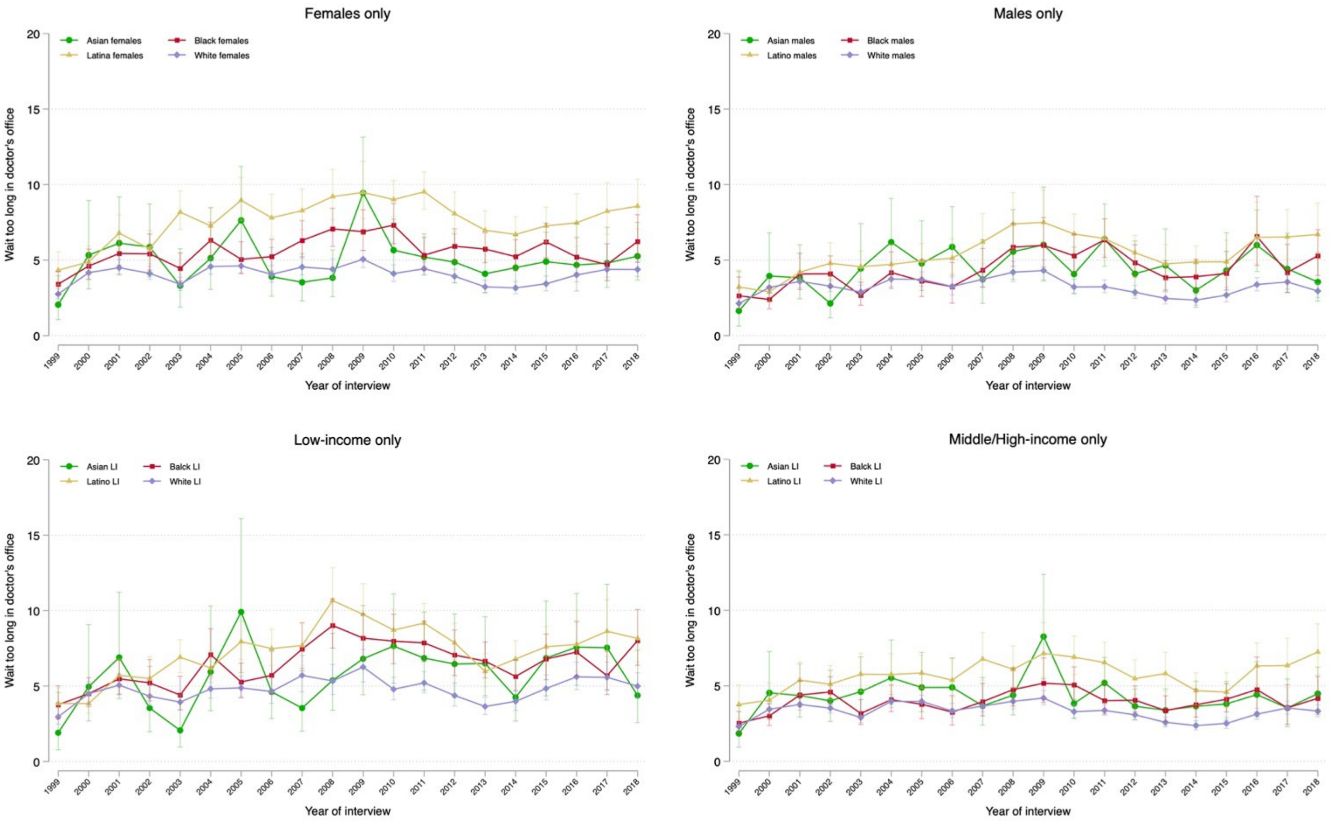

© 2022 Caraballo C et al. *JAMA Health Forum.*

eFigure 7. Trends in Proportion of Individuals Reporting Delaying Care Because the Doctor's Office Was Not Open When They Could Get There by Race and Ethnicity, Stratified by Sex and Income Level

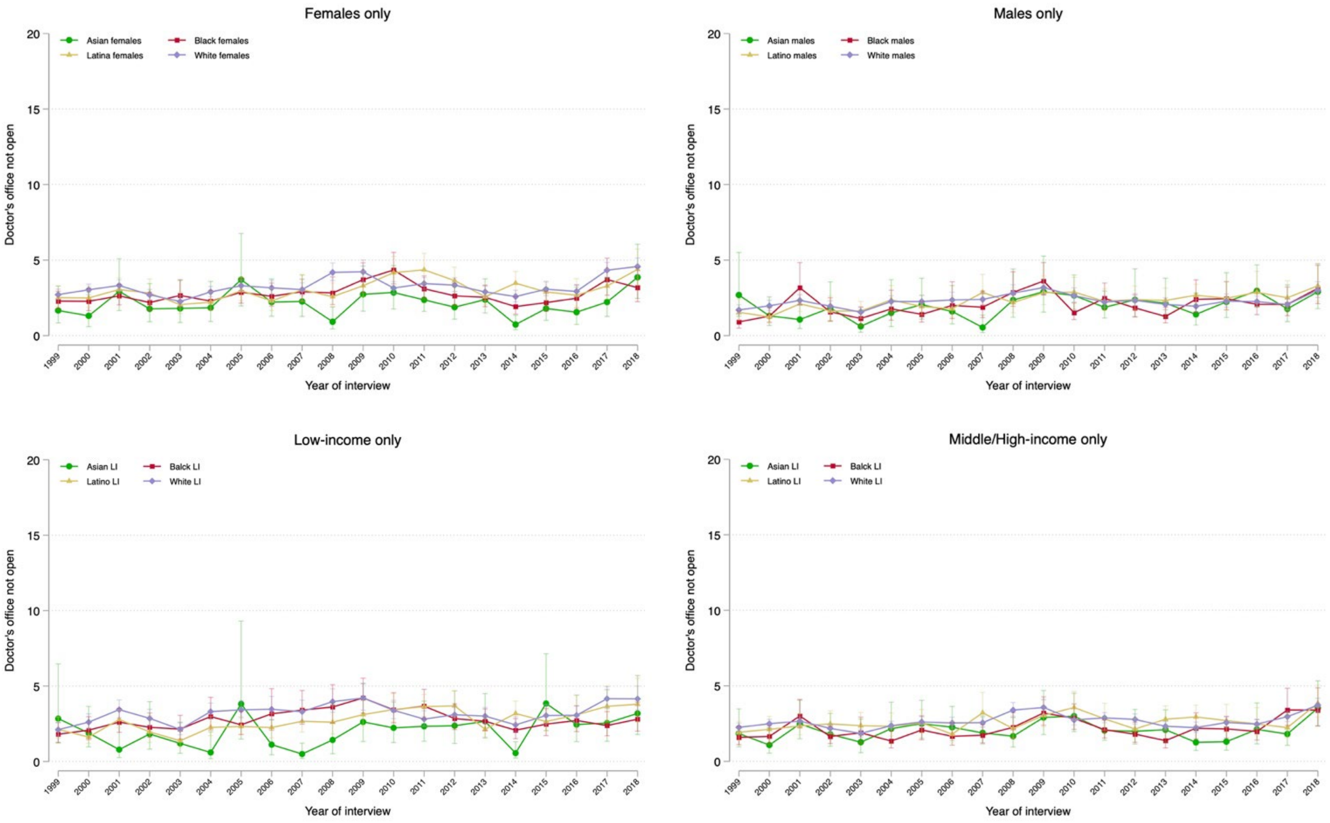

© 2022 Caraballo C et al. *JAMA Health Forum.*

eFigure 8. Trends in Proportion of Individuals Reporting Delaying Care Because They Lacked Transportation by Race and Ethnicity, Stratified by Sex and Income Level

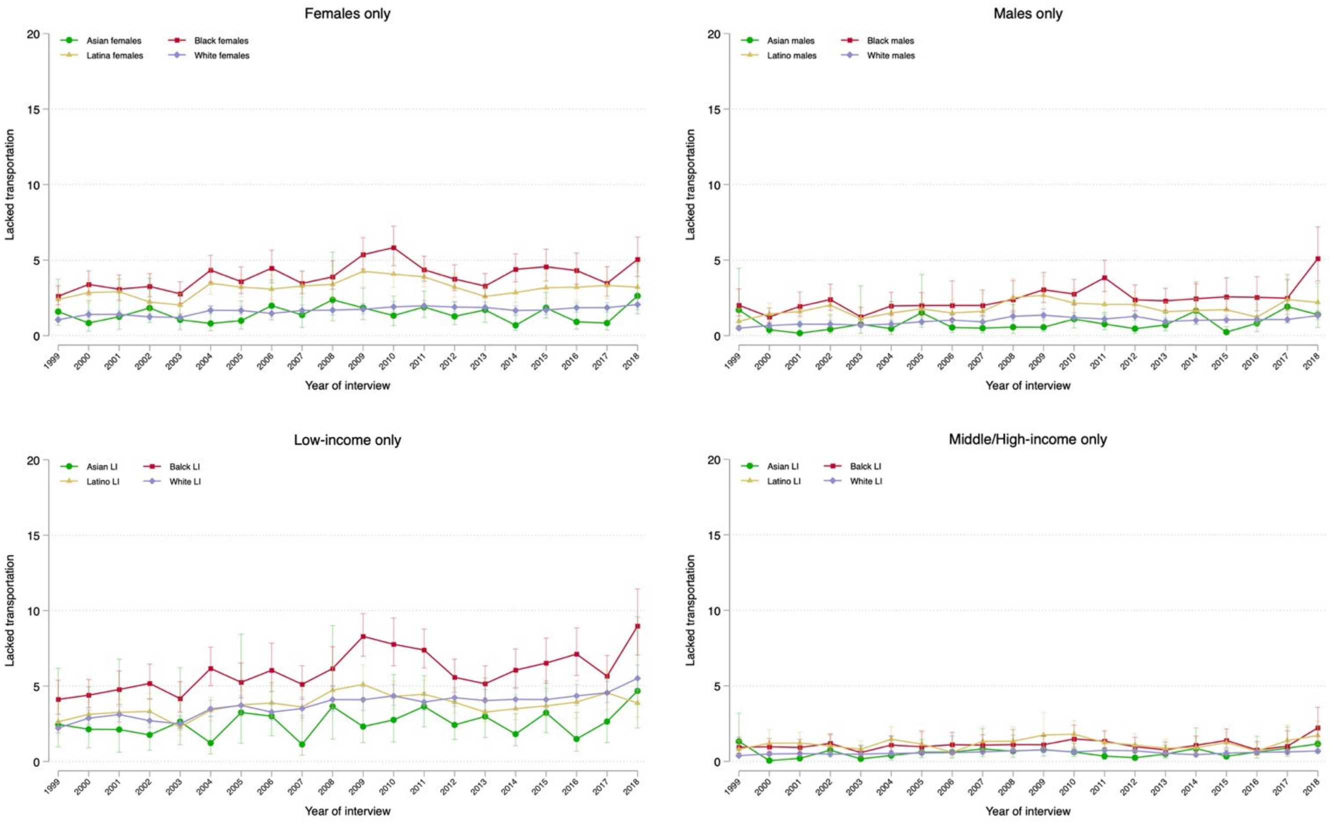

© 2022 Caraballo C et al. *JAMA Health Forum.*

eTable 1. Study Population Characteristics by Years

	Asian			Black			Hispanic/Latino			White		
	**1999–2000**	**2008–2009**	**2017–2018**	**1999–2000**	**2008–2009**	**2017–2018**	**1999–2000**	**2008–2009**	**2017–2018**	**1999–2000**	**2008–2009**	**2017–2018**
Sample size, n [Total=590,603]	n=1572	n=2801	n=2611	n=8639	n=7713	n=5818	n=10,188	n=8754	n=6379	n=41,503	n=29,252	n=36,134
**Age in years**	38 (28–50)	41 (30–55)	43 (32–57)	40 (29–52)	42 (29–55)	43 (30–58)	37 (27–49)	37 (28–50)	39 (28–53)	44 (33–59)	47 (33–61)	50 (34–64)
**Age category**												
18–39 years	52.7 (49.5, 55.9)	46.0 (43.4, 48.7)	41.9 (39.3, 44.6)	49.8 (48.3, 51.3)	45.3 (43.5, 47.1)	43.7 (41.8, 45.7)	56.8 (55.3, 58.2)	54.6 (53.0, 56.2)	50.1 (48.4, 51.9)	39.6 (38.9, 40.2)	34.9 (34.0, 35.8)	33.3 (32.5, 34.1)
40–64 years	37.8 (34.7, 40.9)	40.8 (38.2, 43.5)	42.5 (40.2, 44.9)	38.5 (37.2, 39.9)	42.8 (38.2, 43.5)	41.0 (39.2, 42.9)	34.2 (33.1, 35.3)	37.0 (35.6, 38.4)	39.1 (37.7, 40.6)	42.1 (41.5, 42.7)	45.8 (45.1, 46.6)	42.3 (41.6, 43.0)
≥65 years	9.5 (7.9, 11.4)	13.2 (11.7, 14.8)	15.5 (13.8, 17.3)	11.7 (10.9, 12.6)	12.0 (11.1, 12.9)	15.3 (14.2, 16.3)	9.0 (8.1, 10.0)	8.4 (7.7, 9.1)	10.8 (9.9, 11.7)	18.4 (17.8, 18.9)	19.3 (18.6, 19.9)	24.4 (23.7, 25.1)
**Female**	51.9 (51.4, 52.5)	51.7 (51.0, 52.4)	51.4 (50.7, 52.0)	55.6 (54.1, 57.1)	55.4 (53.9, 56.9)	54.5 (52.8, 56.3)	50.9 (49.6, 52.1)	48.7 (47.2, 50.1)	50.3 (48.7, 52.0)	51.9 (51.4, 52.5)	51.7 (51.0, 52.4)	51.4 (50.7, 52.0)
**US citizenship** [n=589,337]	58.3 (54.7, 61.9)	69.6 (66.9, 72.2)	70.8 (68.1, 73.3)	95.4 (94.7, 96.0)	95.0 (94.2, 95.6)	95.0 (93.8, 95.9)	62.8 (60.7, 64.9)	62.1 (60.1, 64.0)	72.4 (70.7, 74.1)	98.4 (98.2, 98.6)	98.5 (98.3, 98.7)	98.5 (98.3, 98.7)
**Education level** [n=586,373]												
Less than high school	11.4 (9.4, 13.7)	9.2 (7.9, 10.7)	8.0 (6.7, 9.6)	24.4 (23.0, 25.7)	17.6 (16.3, 19.0)	14.2 (12.9, 15.7)	44.5 (42.9, 46.2)	38.6 (37.0, 40.2)	27.8 (26.6, 29.7)	13.4 (12.9, 13.9)	10.5 (10.0, 11.1)	7.4 (6.9, 7.9)
High school diploma /GED	18.1 (16.1, 20.3)	16.2 (14.4, 18.2)	15.3 (13.4, 17.4)	31.4 (30.1, 32.8)	30.7 (29.2, 32.2)	28.9 (27.3, 30.6)	24.3 (23.3, 25.4)	26.6 (25.2, 28.0)	26.6 (25.1, 28.3)	31.7 (31.0, 32.4)	28.2 (27.5, 28.9)	23.6 (22.9, 24.4)
Some college	24.2 (21.7, 27.0)	24.4 (22.2, 26.7)	20.9 (18.9, 23.0)	29.7 (28.4, 31.0)	34.0 (32.5, 35.5)	32.7 (30.9, 34.5)	21.8 (20.6, 23.1)	22.5 (21.4, 23.7)	28.2 (26.6, 29.8)	29.6 (29.1, 30.1)	31.6 (30.9, 32.3)	31.4 (30.6, 32.1)
≥Bachelor's degree	46.2 (43.2, 49.3)	50.2 (46.9, 53.5)	55.9 (52.8, 58.8)	14.5 (13.4, 15.7)	17.8 (16.6, 19.0)	24.2 (22.3, 26.2)	9.4 (8.5, 10.3)	12.4 (11.4, 13.4)	17.4 (16.0, 19.0)	25.4 (24.7, 26.1)	29.8 (28.9, 30.7)	37.6 (36.5, 38.7)
**Income <200% of Federal Poverty Level[a]**	28.2 (24.0, 32.7)	27.0 (23.8, 30.4)	26.3 (22.9, 30.0)	45.5 (43.2, 47.8)	45.0 (42.8, 47.3)	44.5 (41.5, 47.5)	51.0 (48.9, 53.1)	50.2 (48.0, 52.3)	46.7 (44.0, 49.3)	22.9 (22.1, 23.7)	23.9 (22.8, 25.0)	21.3 (20.3, 22.2)
**Uninsured at the time of interview** [n=588,490]	17.3 (15.1, 19.8)	13.9 (12.1, 15.7)	6.3 (5.2, 7.6)	20.2 (19.1, 21.3)	20.8 (19.5, 22.0)	11.9 (10.6, 13.4)	36.1 (34.3, 37.8)	38.9 (37.0, 40.8)	24.1 (22.2, 26.0)	11.0 (10.6, 11.4)	12.3 (11.8, 12.8)	6.5 (6.2, 6.9)
**Region of residence[b]**												
Northeast	21.2 (18.7, 24.0)	18.6 (16.2, 21.3)	20.8 (16.8, 25.3)	17.8 (16.3, 19.4)	16.4 (14.8, 18.1)	16.0 (13.5, 18.8)	15.7 (14.5, 17.0)	13.3 (11.7, 15.1)	13.4 (11.2, 16.1)	20.2 (19.4, 20.9)	18.2 (17.2, 19.2)	19.2 (17.5, 21.1)

© 2022 Caraballo C et al. *JAMA Health Forum.*

	Asian			Black			Hispanic/Latino			White		
	1999–2000	2008–2009	2017–2018	1999–2000	2008–2009	2017–2018	1999–2000	2008–2009	2017–2018	1999–2000	2008–2009	2017–2018
Midwest	14.3 (11.6, 17.6)	15.1 (12.7, 17.8)	11.9 (9.5, 14.7)	18.6 (17.1, 20.3)	19.4 (17.4, 21.5)	15.1 (12.9, 17.7)	8.0 (6.9, 9.2)	9.5 (8.0, 11.2)	9.4 (7.6, 11.6)	29.5 (28.6, 30.3)	28.6 (27.2, 30.0)	27.4 (25.7, 29.3)
South	19.1 (16.5, 22.0)	19.4 (17.2, 21.8)	25.4 (21.4, 29.9)	56.0 (53.6, 58.3)	56.1 (53.5, 58.7)	60.6 (56.8, 64.4)	35.6 (33.4, 37.9)	35.4 (33.3, 37.4)	37.2 (32.6, 42.1)	33.9 (33.0, 34.9)	33.8 (32.3, 35.4)	33.0 (30.8, 35.2)
West	45.4 (41.4, 49.4)	46.9 (43.6, 50.2)	42.0 (36.8, 47.2)	7.6 (6.7, 8.6)	8.2 (7.3, 9.1)	8.3 (6.8, 9.9)	40.8 (38.5, 43.1)	41.9 (39.5, 44.3)	39.9 (35.3, 44.8)	16.5 (15.8, 17.2)	19.4 (18.3, 20.5)	20.4 (18.3, 22.7)
**Married or living with partner** [n=588,349]	64.3 (61.2, 67.3)	64.1 (61.2, 66.9)	65.0 (62.4, 67.4)	37.4 (35.9, 38.9)	35.2 (33.6, 36.8)	32.7 (31.0, 34.4)	58.5 (57.3, 59.6)	54.5 (52.9, 56.1)	49.3 (47.7, 51.0)	61.7 (60.9, 62.4)	58.0 (57.0, 58.9)	56.4 (55.6, 57.1)
**Employment status** [n=589,945]												
With a job/Working	65.9 (63.2, 68.5)	65.1 (62.6, 67.4)	67.3 (65.0, 69.4)	64.2 (62.8, 65.6)	60.8 (59.2, 62.3)	61.1 (59.0, 63.1)	66.3 (65.0, 67.5)	64.1 (62.8, 65.4)	66.6 (64.7, 68.3)	65.7 (65.0, 66.4)	62.6 (61.7, 63.5)	62.1 (61.3, 62.9)
Not in labor force	31.9 (29.3, 34.7)	30.3 (28.0, 32.7)	30.0 (27.7, 32.3)	31.8 (30.4, 33.6)	31.2 (29.8, 32.7)	32.4 (30.5, 34.4)	31.3 (30.1, 32.5)	29.0 (27.9, 30.2)	29.9 (28.0, 31.8)	32.9 (32.3, 33.6)	33.3 (32.4, 34.2)	35.4 (34.7, 36.2)
Unemployed	2.2 (1.5, 3.2)	4.6 (3.7, 5.7)	2.8 (2.1, 3.7)	3.9 (3.4, 4.6)	8.0 (7.2, 8.9)	6.6 (5.7, 7.5)	2.5 (2.1, 2.9)	6.9 (6.1, 7.7)	3.6 (3.0, 4.3)	1.4 (1.2, 1.5)	4.1 (3.8, 4.4)	2.5 (2.2, 2.7)
**Current smoker**	14.9 (12.8, 17.2)	11.0 (9.5, 12.7)	7.2 (6.1, 8.6)	23.9 (22.6, 25.2)	21.3 (20.0, 22.7)	14.8 (13.5, 16.2)	18.4 (17.4, 19.3)	15.1 (14.1, 16.2)	9.8 (8.9, 10.7)	24.2 (23.7, 24.8)	22.1 (21.4, 22.8)	15.1 (14.5, 15.7)
**Obese (BMI ≥30 kg/m^2)**	6.2 (4.9, 7.7)	8.7 (7.2, 10.6)	12.0 (10.6, 13.7)	29.1 (28.0, 30.1)	37.1 (35.8, 38.5)	39.5 (37.9, 41.2)	23.0 (21.9, 24.1)	31.2 (29.9, 32.4)	34.0 (32.5, 35.6)	20.2 (19.7, 20.7)	26.1 (25.4, 26.8)	30.3 (29.6, 31.0)
**Health conditions**												
Asthma	5.6 (4.5, 6.9)	8.9 (7.6, 10.3)	8.5 (7.2, 9.9)	9.0 (8.2, 9.9)	13.8 (12.8, 13.9)	15.1 (14.0, 16.2)	7.1 (6.5, 7.8)	10.0 (9.2, 10.8)	11.7 (10.5, 12.9)	9.2 (8.9, 9.6)	13.4 (12.9, 13.9	13.9 (13.5, 14.4)
Cancer	1.8 (1.1, 2.7)	3.0 (2.5, 3.7)	4.2 (3.4, 5.3)	3.0 (2.6, 3.5)	3.8 (3.3, 4.3)	4.8 (4.3, 5.4)	2.2 (1.8, 2.5)	2.7 (2.3, 3.2)	3.4 (2.9, 3.8)	7.8 (7.6, 8.1)	10.2 (9.8, 10.6)	12.4 (12.0, 12.8)
COPD	1.6 (1.0, 2.4)	1.9 (1.3, 2.7)	2.0 (1.4, 2.7)	4.2 (3.8, 4.7)	4.5 (3.9, 5.1)	4.4 (3.7, 5.1)	3.0 (2.6, 3.4)	2.8 (2.4, 3.3)	2.8 (2.4, 3.4)	6.2 (6.0, 6.5)	6.5 (6.2, 6.9)	5.3 (5.0, 5.6)
Diabetes	3.9 (3.0, 5.1)	7.4 (6.2, 8.8)	9.0 (7.8, 10.4)	8.2 (7.5, 8.9)	11.3 (10.5, 12.3)	11.7 (10.8, 12.7)	6.4 (5.8, 7.0)	8.7 (7.9, 9.5)	10.5 (9.6, 11.4)	5.2 (5.0, 5.4)	8.2 (7.8, 8.6)	9.1 (8.7, 9.5)
Heart disease	4.6 (3.6, 6.0)	5.0 (4.0, 6.2)	6.6 (5.6, 7.7)	8.9 (8.2, 9.7)	9.8 (8.9, 10.7)	9.8 (9.0, 10.7)	6.1 (5.5, 6.7)	6.1 (5.5, 6.7)	6.4 (5.7, 7.2)	12.1 (11.7, 12.4)	13.8 (13.2, 14.3)	14.0 (13.6, 14.5)
Hypertension	14.9 (12.9, 17.2)	22.4 (20.2, 24.7)	24.2 (22.1, 26.3)	29.0 (27.7, 30.3)	35.4 (34.0, 36.9)	37.6 (35.8, 39.3)	15.4 (14.5, 16.3)	19.9 (18.8, 21.1)	22.3 (20.9, 23.7)	23.0 (22.5, 23.5)	30.4 (29.7, 31.1)	32.7 (31.9, 33.4)
Kidney disease	0.8 (0.4, 1.5)	1.2 (0.8, 1.7)	1.8 (1.2, 2.6)	2.0 (1.6, 2.4)	2.2 (1.7, 2.7)	2.6 (2.2, 3.1)	1.6 (1.3, 1.9)	1.8 (1.6, 2.0)	2.0 (1.6, 2.4)	1.3 (1.2, 1.5)	1.8 (1.6, 2.0)	2.3 (2.1, 2.5)
Liver disease	1.1 (0.5, 2.4)	1.3 (0.9, 2.0)	1.6 (1.1, 2.4)	1.0 (0.8, 1.3)	0.9 (0.7, 1.2)	1.2 (0.9, 1.5)	1.0 (0.8, 1.3)	1.8 (1.5, 2.2)	2.4 (2.0, 2.9)	1.0 (0.9, 1.1)	1.5 (1.3, 1.7)	1.8 (1.6, 2.0)

© 2022 Caraballo C et al. *JAMA Health Forum.*

	Asian			Black			Hispanic/Latino			White		
	**1999–2000**	**2008–2009**	**2017–2018**	**1999–2000**	**2008–2009**	**2017–2018**	**1999–2000**	**2008–2009**	**2017–2018**	**1999–2000**	**2008–2009**	**2017–2018**
Stroke	0.8 (0.5, 1.2)	1.2 (0.8, 1.7)	1.8 (1.4, 2.5)	2.8 (2.4, 3.2)	3.3 (2.8, 3.8)	4.0 (3.5, 4.6)	1.1 (0.9, 1.4)	1.6 (1.3, 1.9)	2.1 (1.7, 2.6)	2.2 (2.1, 2.3)	3.0 (2.8, 3.2)	3.3 (3.1, 3.6)

Data are presented as % (95% CI) for categorical variables and median (P25–P75) for continuous variables. All percentages are unadjusted and weighted.

[a] Annual family income was categorized relative to the respective year's Federal Poverty Level from the US Census Bureau into middle/high income (≥200%) and low income (<200%). The weighted proportion of individuals with annual income <200% of the Federal Poverty Level was estimated using multiple imputation.
[b] Based on the Census Bureau-recognized region where the housing unit of the survey participant was located.

Abbreviations: BMI, body mass index; CI, confidence interval; COPD, chronic obstructive pulmonary disease; GED, general equivalency diploma.

© 2022 Caraballo C et al. *JAMA Health Forum.*

eTable 2. Annualized Rate of Change in Prevalence of and Racial Differences in Barriers to Timely Medical Care

**Any barrier to timely medical care**	**Asian individuals**<br>Annualized Rate of Change (95% CI), p value	**Black individuals**<br>Annualized Rate of Change (95% CI), p value	**Hispanic/Latino individuals**<br>Annualized Rate of Change (95% CI), p value	**White individuals**<br>Annualized Rate of Change (95% CI), p value
Prevalence				
1999-2010	+0.22 (+0.02, +0.42), 0.03	+0.53 (+0.34, +0.73), <0.001	+0.63 (+0.53, +0.74), <0.001	+0.28 (+0.16, +0.40), <0.001
2010-2014	-0.58 (-1.10, -0.08), 0.02	-0.61 (-1.20, -0.02), 0.04	-0.86 (-1.17, -0.56), <0.001	-0.56 (-0.93, -0.18), 0.01
2014-2018	+1.23 (+0.50, +1.96), 0.003	+0.94 (+0.06, +1.81), 0.04	+1.21 (+0.74, +1.68), <0.001	+1.09 (+0.59, +1.60), <0.001
Difference with White individuals				
1999-2010	-0.04 (-0.21, +0.12), 0.60	+0.27 (+0.12, +0.43), 0.002	+0.39 (+0.24, +0.53), <0.001	-
2010-2014	-0.03 (-0.45, +0.39), 0.87	-0.08, (-0.55, +0.40), 0.74	-0.34 (-0.77, +0.08), 0.11	-
2014-2018	+0.12 (-0.50, +0.74), 0.68	-0.14 (-0.83, +0.55), 0.67	+0.13 (-0.51, +0.76), 0.42	-

Data source is the National Health Interview Survey from years 1999–2018. For change in prevalence and change in difference: a positive sign (+) means the prevalence of each indicator (or its difference with White people) increased and a negative sign (-) means it decreased. Estimates were adjusted by age and US region. Abbreviations: CI, confidence interval

© 2022 Caraballo C et al. *JAMA Health Forum.*

eTable 3. Annual Prevalence of Ordered Number of Barriers by Race and Ethnicity

Number of Barriers	Year of Interview	Asian Individuals	Black Individuals	Hispanic/Latino Individuals	White Individuals
≥1	1999	7.26 (7.24, 7.28)	7.02 (6.99, 7.04)	7.85 (7.82, 7.88)	7.10 (7.08, 7.13)
	2000	8.81 (8.79, 8.83)	8.15 (8.12, 8.18)	7.55 (7.52, 7.58)	9.19 (9.16, 9.21)
	2001	10.23 (10.20, 10.25)	11.14 (11.10, 11.17)	9.94 (9.90, 9.97)	10.07 (10.04, 10.10)
	2002	9.61 (9.58, 9.63)	9.75 (9.71, 9.79)	9.18 (9.15, 9.21)	8.97 (8.94, 9.00)
	2003	8.07 (8.05, 8.09)	8.50 (8.46, 8.53)	10.41 (10.37, 10.44)	7.73 (7.71, 7.76)
	2004	10.22 (10.19, 10.25)	10.75 (10.71, 10.79)	10.58 (10.55, 10.62)	9.67 (9.64, 9.70)
	2005	10.14 (10.11, 10.16)	9.45 (9.41, 9.48)	11.54 (11.51, 11.58)	9.53 (9.50, 9.56)
	2006	9.36 (9.33, 9.39)	9.26 (9.22, 9.31)	10.88 (10.83, 10.93)	9.34 (9.31, 9.38)
	2007	7.46 (7.43, 7.49)	10.04 (9.99, 10.10)	12.13 (12.08, 12.18)	10.09 (10.05, 10.13)
	2008	9.75 (9.71, 9.78)	12.56 (12.50, 12.62)	14.29 (14.23, 14.35)	11.17 (11.12, 11.22)
	2009	13.15 (13.11, 13.19)	14.86 (14.80, 14.92)	14.67 (14.62, 14.73)	11.78 (11.74, 11.82)
	2010	10.26 (10.23, 10.29)	14.69 (14.63, 14.75)	14.58 (14.53, 14.64)	10.28 (10.24, 10.31)
	2011	11.38 (11.35, 11.41)	13.22 (13.18, 13.27)	14.33 (14.28, 14.38)	10.67 (10.64, 10.70)
	2012	9.75 (9.73, 9.78)	11.83 (11.79, 11.87)	12.78 (12.73, 12.82)	10.23 (10.20, 10.26)
	2013	8.70 (8.68, 8.72)	10.91 (10.87, 10.95)	11.43 (11.39, 11.47)	8.83 (8.80, 8.86)
	2014	7.32 (7.30, 7.34)	10.78 (10.74, 10.83)	11.78 (11.74, 11.82)	8.81 (8.78, 8.84)
	2015	10.27 (10.25, 10.30)	12.62 (12.57, 12.66)	11.92 (11.88, 11.96)	10.02 (9.98, 10.05)
	2016	10.96 (10.91, 11.02)	13.16 (13.09, 13.23)	13.89 (13.82, 13.95)	11.01 (10.95, 11.06)
	2017	11.34 (11.27, 11.40)	12.42 (12.35, 12.50)	14.74 (14.67, 14.81)	12.10 (12.02, 12.17)
	2018	13.06 (13.00, 13.12)	14.90 (14.82, 14.98)	15.99 (15.93, 16.05)	12.98 (12.91, 13.05)
≥2	1999	2.66 (2.66, 2.67)	2.82 (2.81, 2.83)	3.28 (3.27, 3.29)	2.70 (2.69, 2.71)
	2000	3.27 (3.26, 3.28)	3.30 (3.29, 3.31)	3.15 (3.14, 3.16)	3.55 (3.54, 3.56)
	2001	3.83 (3.82, 3.84)	4.61 (4.59, 4.62)	4.21 (4.20, 4.23)	3.91 (3.90, 3.92)
	2002	3.59 (3.58, 3.60)	4.00 (3.98, 4.01)	3.87 (3.86, 3.88)	3.46 (3.45, 3.47)
	2003	2.98 (2.97, 2.99)	3.45 (3.44, 3.46)	4.42 (4.41, 4.44)	2.96 (2.95, 2.97)
	2004	3.83 (3.82, 3.84)	4.43 (4.41, 4.45)	4.51 (4.49, 4.52)	3.74 (3.73, 3.76)
	2005	3.80 (3.79, 3.81)	3.86 (3.85, 3.88)	4.95 (4.93, 4.96)	3.69 (3.68, 3.70)
	2006	3.49 (3.47, 3.50)	3.78 (3.76, 3.80)	4.64 (4.62, 4.66)	3.61 (3.60, 3.63)
	2007	2.74 (2.73, 2.75)	4.12 (4.10, 4.15)	5.22 (5.19, 5.24)	3.92 (3.90, 3.94)

© 2022 Caraballo C et al. *JAMA Health Forum.*

Number of Barriers	Year of Interview	Asian Individuals	Black Individuals	Hispanic/Latino Individuals	White Individuals
	2008	3.64 (3.63, 3.66)	5.25 (5.22, 5.27)	6.24 (6.21, 6.27)	4.37 (4.35, 4.39)
	2009	5.04 (5.02, 5.05)	6.31 (6.28, 6.34)	6.42 (6.40, 6.45)	4.63 (4.61, 4.65)
	2010	3.85 (3.84, 3.86)	6.23 (6.20, 6.26)	6.38 (6.35, 6.41)	4.00 (3.98, 4.01)
	2011	4.30 (4.29, 4.31)	5.55 (5.53, 5.57)	6.26 (6.24, 6.28)	4.16 (4.15, 4.18)
	2012	3.64 (3.63, 3.65)	4.92 (4.90, 4.94)	5.52 (5.50, 5.54)	3.98 (3.97, 3.99)
	2013	3.23 (3.22, 3.24)	4.50 (4.49, 4.52)	4.89 (4.88, 4.91)	3.40 (3.39, 3.41)
	2014	2.69 (2.68, 2.70)	4.45 (4.43, 4.47)	5.05 (5.04, 5.07)	3.39 (3.38, 3.40)
	2015	3.85 (3.84, 3.86)	5.27 (5.25, 5.29)	5.12 (5.10, 5.14)	3.89 (3.88, 3.90)
	2016	4.13 (4.11, 4.15)	5.52 (5.49, 5.55)	6.05 (6.02, 6.08)	4.30 (4.28, 4.33)
	2017	4.28 (4.26, 4.31)	5.18 (5.15, 5.22)	6.45 (6.42, 6.49)	4.77 (4.74, 4.80)
	2018	5.00 (4.97, 5.02)	6.33 (6.29, 6.36)	7.06 (7.03, 7.09)	5.15 (5.12, 5.18)
≥3	1999	0.95 (0.95, 0.95)	1.22 (1.21, 1.22)	1.38 (1.38, 1.39)	1.04 (1.03, 1.04)
	2000	1.17 (1.17, 1.17)	1.43 (1.43, 1.44)	1.33 (1.32, 1.33)	1.37 (1.36, 1.37)
	2001	1.38 (1.37, 1.38)	2.01 (2.01, 2.02)	1.79 (1.78, 1.79)	1.51 (1.51, 1.51)
	2002	1.29 (1.28, 1.29)	1.74 (1.73, 1.75)	1.64 (1.63, 1.64)	1.33 (1.33, 1.34)
	2003	1.06 (1.06, 1.07)	1.50 (1.49, 1.50)	1.88 (1.87, 1.89)	1.13 (1.13, 1.14)
	2004	1.38 (1.37, 1.38)	1.94 (1.93, 1.94)	1.91 (1.91, 1.92)	1.44 (1.44, 1.45)
	2005	1.36 (1.36, 1.37)	1.68 (1.67, 1.69)	2.11 (2.10, 2.11)	1.42 (1.42, 1.43)
	2006	1.25 (1.24, 1.25)	1.64 (1.64, 1.65)	1.97 (1.96, 1.98)	1.39 (1.39, 1.40)
	2007	0.98 (0.97, 0.98)	1.80 (1.79, 1.81)	2.23 (2.22, 2.24)	1.51 (1.51, 1.52)
	2008	1.31 (1.30, 1.31)	2.30 (2.29, 2.32)	2.68 (2.67, 2.69)	1.69 (1.69, 1.70)
	2009	1.82 (1.82, 1.83)	2.79 (2.77, 2.80)	2.76 (2.75, 2.77)	1.80 (1.79, 1.80)
	2010	1.38 (1.38, 1.39)	2.75 (2.74, 2.76)	2.74 (2.73, 2.75)	1.54 (1.54, 1.55)
	2011	1.55 (1.55, 1.55)	2.44 (2.43, 2.45)	2.69 (2.68, 2.70)	1.61 (1.60, 1.62)
	2012	1.31 (1.30, 1.31)	2.15 (2.14, 2.16)	2.36 (2.35, 2.37)	1.54 (1.53, 1.54)
	2013	1.15 (1.15, 1.16)	1.97 (1.96, 1.98)	2.08 (2.08, 2.09)	1.31 (1.30, 1.31)
	2014	0.96 (0.95, 0.96)	1.94 (1.93, 1.95)	2.15 (2.15, 2.16)	1.30 (1.30, 1.31)
	2015	1.38 (1.38, 1.39)	2.31 (2.31, 2.32)	2.18 (2.17, 2.19)	1.50 (1.50, 1.51)
	2016	1.49 (1.48, 1.49)	2.43 (2.41, 2.44)	2.59 (2.58, 2.61)	1.67 (1.66, 1.68)
	2017	1.54 (1.53, 1.55)	2.27 (2.26, 2.29)	2.78 (2.76, 2.79)	1.85 (1.84, 1.86)
	2018	1.81 (1.80, 1.82)	2.80 (2.78, 2.81)	3.05 (3.04, 3.06)	2.00 (1.99, 2.02)
≥4	1999	0.25 (0.25, 0.25)	0.41 (0.41, 0.42)	0.46 (0.46, 0.46)	0.32 (0.32, 0.32)

© 2022 Caraballo C et al. *JAMA Health Forum.*

Number of Barriers	Year of Interview	Asian Individuals	Black Individuals	Hispanic/Latino Individuals	White Individuals
	2000	0.30 (0.30, 0.30)	0.49 (0.48, 0.49)	0.44 (0.44, 0.45)	0.43 (0.43, 0.43)
	2001	0.36 (0.36, 0.36)	0.69 (0.68, 0.69)	0.60 (0.60, 0.60)	0.47 (0.47, 0.47)
	2002	0.33 (0.33, 0.34)	0.59 (0.59, 0.59)	0.55 (0.55, 0.55)	0.42 (0.41, 0.42)
	2003	0.28 (0.28, 0.28)	0.51 (0.51, 0.51)	0.63 (0.63, 0.63)	0.35 (0.35, 0.36)
	2004	0.36 (0.36, 0.36)	0.66 (0.66, 0.66)	0.64 (0.64, 0.65)	0.45 (0.45, 0.45)
	2005	0.35 (0.35, 0.36)	0.57 (0.57, 0.57)	0.71 (0.71, 0.71)	0.44 (0.44, 0.45)
	2006	0.32 (0.32, 0.33)	0.56 (0.56, 0.56)	0.66 (0.66, 0.67)	0.44 (0.43, 0.44)
	2007	0.25 (0.25, 0.25)	0.61 (0.61, 0.62)	0.75 (0.75, 0.75)	0.47 (0.47, 0.48)
	2008	0.34 (0.34, 0.34)	0.79 (0.78, 0.79)	0.90 (0.90, 0.91)	0.53 (0.53, 0.53)
	2009	0.48 (0.47, 0.48)	0.96 (0.95, 0.96)	0.93 (0.93, 0.94)	0.56 (0.56, 0.57)
	2010	0.36 (0.36, 0.36)	0.94 (0.94, 0.95)	0.93 (0.92, 0.93)	0.48 (0.48, 0.49)
	2011	0.40 (0.40, 0.40)	0.83 (0.83, 0.84)	0.91 (0.90, 0.91)	0.50 (0.50, 0.51)
	2012	0.34 (0.34, 0.34)	0.73 (0.73, 0.74)	0.80 (0.79, 0.80)	0.48 (0.48, 0.48)
	2013	0.30 (0.30, 0.30)	0.67 (0.67, 0.67)	0.70 (0.70, 0.70)	0.41 (0.41, 0.41)
	2014	0.25 (0.25, 0.25)	0.66 (0.66, 0.67)	0.72 (0.72, 0.73)	0.41 (0.41, 0.41)
	2015	0.36 (0.36, 0.36)	0.79 (0.79, 0.79)	0.73 (0.73, 0.74)	0.47 (0.47, 0.47)
	2016	0.39 (0.39, 0.39)	0.83 (0.82, 0.84)	0.88 (0.87, 0.88)	0.52 (0.52, 0.53)
	2017	0.40 (0.40, 0.40)	0.78 (0.77, 0.78)	0.94 (0.93, 0.94)	0.58 (0.58, 0.59)
	2018	0.47 (0.47, 0.47)	0.96 (0.95, 0.96)	1.03 (1.03, 1.04)	0.63 (0.63, 0.63)
≥5	1999	0.03 (0.03, 0.03)	0.10 (0.10, 0.10)	0.11 (0.11, 0.11)	0.07 (0.07, 0.07)
	2000	0.04 (0.04, 0.04)	0.12 (0.12, 0.12)	0.11 (0.11, 0.11)	0.09 (0.09, 0.09)
	2001	0.05 (0.05, 0.05)	0.17 (0.17, 0.17)	0.15 (0.15, 0.15)	0.10 (0.10, 0.10)
	2002	0.05 (0.05, 0.05)	0.15 (0.15, 0.15)	0.14 (0.14, 0.14)	0.08 (0.08, 0.09)
	2003	0.04 (0.04, 0.04)	0.13 (0.13, 0.13)	0.16 (0.16, 0.16)	0.07 (0.07, 0.07)
	2004	0.05 (0.05, 0.05)	0.17 (0.16, 0.17)	0.16 (0.16, 0.16)	0.09 (0.09, 0.09)
	2005	0.05 (0.05, 0.05)	0.14 (0.14, 0.14)	0.18 (0.18, 0.18)	0.09 (0.09, 0.09)
	2006	0.05 (0.05, 0.05)	0.14 (0.14, 0.14)	0.16 (0.16, 0.17)	0.09 (0.09, 0.09)
	2007	0.04 (0.04, 0.04)	0.15 (0.15, 0.15)	0.19 (0.19, 0.19)	0.10 (0.10, 0.10)
	2008	0.05 (0.05, 0.05)	0.20 (0.20, 0.20)	0.22 (0.22, 0.23)	0.11 (0.11, 0.11)
	2009	0.07 (0.07, 0.07)	0.24 (0.24, 0.24)	0.23 (0.23, 0.23)	0.12 (0.11, 0.12)
	2010	0.05 (0.05, 0.05)	0.24 (0.24, 0.24)	0.23 (0.23, 0.23)	0.10 (0.10, 0.10)
	2011	0.06 (0.06, 0.06)	0.21 (0.21, 0.21)	0.23 (0.22, 0.23)	0.10 (0.10, 0.10)

© 2022 Caraballo C et al. *JAMA Health Forum.*

Number of Barriers	Year of Interview	Asian Individuals	Black Individuals	Hispanic/Latino Individuals	White Individuals
	2012	0.05 (0.05, 0.05)	0.18 (0.18, 0.19)	0.20 (0.20, 0.20)	0.10 (0.10, 0.10)
	2013	0.04 (0.04, 0.04)	0.17 (0.17, 0.17)	0.17 (0.17, 0.17)	0.08 (0.08, 0.08)
	2014	0.03 (0.03, 0.03)	0.17 (0.17, 0.17)	0.18 (0.18, 0.18)	0.08 (0.08, 0.08)
	2015	0.05 (0.05, 0.05)	0.20 (0.20, 0.20)	0.18 (0.18, 0.18)	0.10 (0.10, 0.10)
	2016	0.05 (0.05, 0.05)	0.21 (0.21, 0.21)	0.22 (0.22, 0.22)	0.11 (0.11, 0.11)
	2017	0.06 (0.06, 0.06)	0.19 (0.19, 0.20)	0.23 (0.23, 0.23)	0.12 (0.12, 0.12)
	2018	0.07 (0.07, 0.07)	0.24 (0.24, 0.24)	0.26 (0.26, 0.26)	0.13 (0.13, 0.13)

© 2022 Caraballo C et al. *JAMA Health Forum.*

eTable 4. Change in the Adjusted Prevalence of Any Barriers to Timely Medical Care Access from 1999 to 2018, by Race and Ethnicity and Stratified by Sex and Income Level

	**Asian individuals**	**Black individuals**	**Hispanic/Latino individuals**	**White individuals**
	Percentage points (95% CI), p value	Percentage points (95% CI), p value	Percentage points (95% CI), p value	Percentage points (95% CI), p value
Absolute change in prevalence, 1999–2018				
Females	+7.38 (+2.68, +12.08), 0.002	+7.68 (+4.96, +10.40), <0.001	+8.52 (+5.83, +11.20), <0.001	+6.77 (+5.64, +7.91), <0.001
Males	+3.89 (-0.63, +8.42), 0.09	+8.50 (+5.45, +11.54), <0.001	+7.65 (+4.68, +10.63), <0.001	+4.93 (+3.93, +5.93), <0.001
Low-income	+10.22 (+2.47, +17.97), 0.01	+10.16 (+6.80, +13.52), <0.001	+8.95 (+5.91, +11.99), <0.001	+9.07 (+7.37, +10.77), <0.001
Middle/high-income	+4.20 (+0.65, +7.76), 0.02	+6.42 (+3.97, +8.87), <0.001	+7.26 (+4.47, +10.04), <0.001	+5.02 (+4.16, +5.88), <0.001
Difference with White, 1999				-
Females	-1.22 (-3.92, +1.48), 0.38	-0.03 (-1.39, +1.32), 0.96	+0.78 (-0.70, +2.27), 0.30	-
Males	+1.75 (-1.53, +5.04), 0.30	-0.35 (-1.89, +1.19), 0.66	+0.99 (-0.69, +2.67), 0.25	-
Low-income	-0.65 (-4.63, +3.33), 0.75	+0.75 (-1.07, +2.57), 0.42	-0.16 (-1.87, +1.56), 0.86	-
Middle/high-income	+0.42 (-2.04, +2.88), 0.74	-1.16 (-2.48, +0.16), 0.09	+1.27 (-0.31, +2.86), 0.12	-
Difference with White, 2018				-
Females	-0.62 (-4.63, +3.40), 0.76	+0.87 (-1.74, +3.49), 0.51	+2.53 (+0.02, +5.03), 0.05	-
Males	+0.72 (-2.55, +3.98), 0.67	+3.22 (+0.41, +6.03), 0.03	+3.72 (+1.07, +6.37), 0.01	-
Low-income	+0.50 (-6.37, +7.36), 0.89	+1.84 (-1.46, +5.14), 0.27	-0.28 (-3.31, +2.76), 0.86	-
Middle/high-income	-0.39 (-3.09, +2.31), 0.78	+0.25 (-1.99, +2.48), 0.83	+3.52 (+1.07, +5.96), 0.01	-
Change in difference with White, 1999–2018				-
Females	+0.60 (-4.23, +5.44), 0.81	+0.91 (-2.04, +3.85), 0.55	+1.75 (-1.17, +4.66), 0.24	-
Males	-1.04 (-5.67, +3.60), 0.66	+3.57 (+0.36, +6.77), 0.03	+2.73 (-0.41, +5.86), 0.09	-
Low-income	+1.15 (-6.79, +9.08), 0.78	+1.09 (-2.68, +4.86), 0.57	-0.12 (-3.60, +3.36), 0.95	-
Middle/high-income	-0.81 (-4.47, +2.84), 0.66	+1.40 (-1.19, +4.00), 0.29	+2.24 (-0.67, +5.16), 0.13	-

Data source is the National Health Interview Survey from years 1999 to 2018. For change in prevalence and change in difference: a positive sign (+) means the prevalence of any barrier to timely medical care (or its difference with White people) increased and a negative sign (-) means it decreased. Estimates were adjusted by age and US region.
Abbreviations: CI, confidence interval

eTable 5. Sensitivity Analysis: Change in the Adjusted Prevalence of Any Barriers to Timely Medical Care Access from 1999 to 2018, by Race and Ethnicity and Stratified by Insurance Status and Presence of Affordability Barriers To Care

	**Asian individuals**	**Black individuals**	**Hispanic/Latino individuals**	**White individuals**
	Percentage points (95% CI), p value	Percentage points (95% CI), p value	Percentage points (95% CI), p value	Percentage points (95% CI), p value
Absolute change in prevalence, 1999–2018				
Uninsured	+2.81 (-6.33, +11.96), 0.55	+7.55 (+2.09, +13.02), 0.007	+6.19 (+2.29, +10.09), 0.002	+0.92 (-1.53, +3.38), 0.46
Insured	+5.69 (+2.03, +9.34), 0.002	+8.27 (+6.17, +10.37), <0.001	+8.45 (+6.14, +10.75), <0.001	+6.27 (+5.45, +7.09), <0.001
With affordability barriers	+5.60 (-7.93, +19.13), 0.42	+15.43 (+9.13, +21.74), <0.001	+13.30 (+7.28, +19.32), <0.001	+8.96 (+6.18, +11.73), <0.001
Without affordability barriers	+5.60 (+2.22, +8.99), 0.001	+5.94 (+4.02, +7.86), <0.001	+6.90 (+4.78, +9.02), <0.001	+4.93 (+4.16, +5.70), <0.001
Difference with White, 1999				-
Uninsured	-2.52 (-7.96, +2.92), 0.36	+0.58 (-2.21, +3.37), 0.68	-0.72 (-2.92, +1.48), 0.52	-
Insured	+0.81 (-1.50, +3.12), 0.49	-0.16 (-1.29, +0.97), 0.78	+1.37 (+0.09, +2.65), 0.04	-
With affordability barriers	+3.60 (-6.08, +13.28), 0.47	+0.24 (-3.67, +4.15), 0.91	+0.88 (-3.12, +4.88), 0.67	-
Without affordability barriers	+0.58 (-1.50, +2.66), 0.59	-0.45 (-1.52, +0.63), 0.41	+0.75 (-0.36, +1.87), 0.19	-
Difference with White, 2018				-
Uninsured	-0.63 (-8.38, +7.12), 0.87	+7.21 (+1.91, +12.52), 0.008	+4.54 (+0.49, +8.59), 0.03	-
Insured	+0.22 (-2.73, +3.17), 0.88	+1.84 (-0.12, +3.79), 0.07	+3.54 (+1.46, +5.63), <0.001	-
With affordability barriers	+0.24 (-9.61, +10.09), 0.96	+6.72 (+1.05, +12.38), 0.02	+5.23 (-0.05, +10.52), 0.05	-
Without affordability barriers	+1.25 (-1.53, +4.03), 0.38	+0.56 (-1.21, +2.33), 0.53	+2.73 (+0.77, +4.68), 0.006	-
Change in difference with White, 1999–2018				-
Uninsured	+1.89 (-7.58, +11.36), 0.70	+6.63 (+0.64, +12.62), 0.03	+5.26 (+0.65, +9.87), 0.03	-
Insured	-0.58 (-4.33, +3.16), 0.76	+2.00 (-0.26, +4.26), 0.08	+2.18 (-0.27, +4.62), 0.08	-
With affordability barriers	-3.36 (-17.17, +10.45) 0.63	+6.48 (-0.41, +13.36), 0.07	+4.35 (-2.28, +10.97), 0.20	-
Without affordability barriers	+0.67 (-2.80, +4.15), 0.71	+1.01 (-1.06, +3.08), 0.34	+1.97 (-0.28, +4.23), 0.09	-

© 2022 Caraballo C et al. *JAMA Health Forum.*

Data source is the National Health Interview Survey from years 1999 to 2018. For change in prevalence and change in difference: a positive sign (+) means the prevalence of any barrier to timely medical care (or its difference with White people) increased and a negative sign (-) means it decreased. Estimates were adjusted by age and US region. Presence of affordability barriers was defined as reporting foregoing or delaying medical care due to cost in the past 12 months. Abbreviations: CI, confidence interval

© 2022 Caraballo C et al. *JAMA Health Forum.*

eTable 6. Annual Estimates (95% CI) of Each Barrier to Timely Medical Care by Race and Ethnicity

A) Any barrier

Year of Interview	Asian Individuals	Black Individuals	Hispanic/Latino Individuals	White Individuals
1999	7.26 (5.55, 9.53)	7.01 (6.04, 8.11)	7.93 (6.97, 9.02)	7.04 (6.64, 7.47)
2000	8.82 (6.79, 11.31)	8.13 (7.29, 9.09)	7.58 (6.73, 8.55)	9.08 (8.58, 9.62)
2001	10.18 (8.08, 12.75)	11.19 (9.98, 12.51)	9.97 (9.03, 11.01)	9.97 (9.43, 10.55)
2002	9.44 (7.60, 11.64)	9.78 (8.65, 11.06)	9.25 (8.16, 10.44)	8.92 (8.45, 9.42)
2003	8.16 (6.20, 10.56)	8.55 (7.50, 9.70)	10.48 (9.51, 11.54)	7.68 (7.23, 8.15)
2004	10.03 (8.05, 12.43)	10.76 (9.67, 11.97)	10.58 (9.60, 11.67)	9.60 (9.07, 10.16)
2005	9.84 (7.73, 12.45)	9.44 (8.47, 10.56)	11.59 (10.53, 12.77)	9.44 (8.93, 9.99)
2006	9.33 (7.67, 11.27)	9.23 (8.07, 10.62)	10.90 (9.69, 12.23)	9.24 (8.60, 9.93)
2007	7.44 (5.99, 9.26)	10.01 (8.84, 11.32)	12.04 (10.70, 13.53)	9.99 (9.40, 10.60)
2008	9.76 (8.06, 11.79)	12.52 (11.16, 13.98)	14.33 (12.80, 15.99)	11.04 (10.28, 11.88)
2009	12.90 (10.18, 16.22)	14.88 (13.41, 16.47)	14.59 (13.01, 16.39)	11.65 (10.98, 12.35)
2010	10.25 (8.58, 12.17)	14.70 (13.44, 16.02)	14.58 (13.37, 15.87)	10.16 (9.61, 10.76)
2011	11.36 (9.83, 13.14)	13.34 (12.15, 14.65)	14.29 (13.19, 15.50)	10.61 (10.07, 11.16)
2012	9.81 (8.32, 11.60)	11.90 (10.75, 13.12)	12.81 (11.53, 14.21)	10.15 (9.59, 10.75)
2013	8.66 (7.41, 10.13)	10.95 (9.82, 12.18)	11.44 (10.43, 12.56)	8.76 (8.27, 9.27)
2014	7.31 (5.88, 9.04)	10.83 (9.76, 12.00)	11.74 (10.72, 12.85)	8.75 (8.18, 9.33)
2015	10.27 (8.56, 12.19)	12.69 (11.37, 14.11)	11.90 (10.73, 13.15)	9.97 (9.39, 10.56)
2016	11.07 (9.07, 13.51)	13.36 (11.69, 15.24)	13.91 (12.40, 15.55)	10.95 (10.36, 11.56)
2017	11.32 (9.27, 13.68)	12.59 (11.03, 14.32)	14.90 (13.27, 16.71)	12.03 (11.38, 12.73)
2018	13.01 (10.58, 15.95)	14.96 (13.35, 16.75)	15.99 (14.31, 17.83)	12.89 (12.23, 13.57)

B) Couldn't get through by phone

Year of Interview	Asian Individuals	Black Individuals	Hispanic/Latino Individuals	White Individuals
1999	2.17 (1.26, 3.75)	1.59 (1.24, 2.04)	1.78 (1.38, 2.30)	2.18 (1.95, 2.43)
2000	2.11 (1.35, 3.26)	1.78 (1.32, 2.37)	1.90 (1.52, 2.38)	2.36 (2.13, 2.62)
2001	2.13 (1.51, 3.01)	2.73 (2.15, 3.50)	2.59 (2.12, 3.15)	2.68 (2.44, 2.95)
2002	1.83 (1.18, 2.86)	1.96 (1.49, 2.56)	2.18 (1.69, 2.80)	2.27 (2.04, 2.54)
2003	0.84 (0.43, 1.66)	1.96 (1.51, 2.53)	2.32 (1.85, 2.91)	1.89 (1.66, 2.14)
2004	2.81 (1.88, 4.15)	2.72 (2.21, 3.34)	2.79 (2.26, 3.45)	2.26 (2.02, 2.52)
2005	2.06 (1.33, 3.17)	1.84 (1.44, 2.34)	2.29 (1.87, 2.78)	2.29 (2.03, 2.58)
2006	1.98 (1.23, 3.18)	2.09 (1.58, 2.75)	2.44 (1.92, 3.13)	2.33 (2.01, 2.72)
2007	1.23 (0.68, 2.23)	2.17 (1.70, 2.77)	2.99 (2.37, 3.75)	2.26 (2.00, 2.55)
2008	0.93 (0.56, 1.52)	2.70 (2.08, 3.50)	2.75 (2.12, 3.56)	2.47 (2.16, 2.81)
2009	3.00 (1.94, 4.61)	3.47 (2.75, 4.38)	3.90 (3.13, 4.84)	2.54 (2.26, 2.85)
2010	1.64 (1.05, 2.59)	3.68 (2.99, 4.50)	2.54 (2.04, 3.16)	2.15 (1.89, 2.43)
2011	1.80 (1.31, 2.46)	2.54 (2.01, 3.20)	3.20 (2.62, 3.88)	2.23 (1.99, 2.49)
2012	1.32 (0.84, 2.08)	2.76 (2.23, 3.41)	2.92 (2.43, 3.51)	2.24 (1.98, 2.53)
2013	1.77 (1.18, 2.64)	2.41 (1.93, 2.99)	2.41 (1.98, 2.93)	1.87 (1.65, 2.13)
2014	1.22 (0.77, 1.91)	2.58 (2.09, 3.16)	2.47 (2.05, 2.98)	2.08 (1.74, 2.48)
2015	1.76 (1.22, 2.50)	2.64 (2.04, 3.42)	2.36 (1.92, 2.90)	2.31 (2.06, 2.58)
2016	2.19 (1.50, 3.20)	2.53 (2.01, 3.17)	3.10 (2.30, 4.21)	2.52 (2.28, 2.79)

© 2022 Caraballo C et al. *JAMA Health Forum.*

Year of Interview	Asian Individuals	Black Individuals	Hispanic/Latino Individuals	White Individuals
2017	2.81 (1.91, 4.08)	2.41 (1.82, 3.17)	3.25 (2.48, 4.23)	2.78 (2.51, 3.09)
2018	2.86 (2.01, 4.10)	3.02 (2.34, 3.92)	3.46 (2.71, 4.41)	3.06 (2.75, 3.40)

C) Couldn't get an appointment soon enough

Year of Interview	Asian Individuals	Black Individuals	Hispanic/Latino Individuals	White Individuals
1999	3.78 (2.60, 5.47)	2.90 (2.34, 3.57)	4.06 (3.40, 4.84)	3.78 (3.50, 4.08)
2000	3.91 (2.80, 5.40)	4.26 (3.62, 5.00)	4.03 (3.44, 4.72)	5.47 (5.10, 5.86)
2001	5.28 (3.79, 7.31)	5.68 (4.93, 6.57)	5.04 (4.32, 5.87)	5.78 (5.37, 6.21)
2002	6.00 (4.50, 7.98)	4.76 (4.07, 5.58)	4.53 (3.85, 5.33)	5.15 (4.80, 5.53)
2003	4.19 (2.90, 5.97)	4.10 (3.39, 4.96)	4.80 (4.05, 5.65)	4.51 (4.18, 4.86)
2004	5.80 (4.34, 7.73)	4.97 (4.27, 5.78)	4.54 (3.92, 5.27)	4.96 (4.60, 5.35)
2005	5.53 (4.02, 7.56)	4.35 (3.69, 5.11)	4.87 (4.19, 5.64)	4.84 (4.49, 5.22)
2006	4.16 (3.13, 5.50)	4.16 (3.46, 5.02)	4.97 (4.20, 5.85)	5.07 (4.62, 5.58)
2007	3.45 (2.45, 4.82)	4.62 (3.89, 5.46)	5.41 (4.39, 6.62)	5.50 (5.08, 5.94)
2008	5.03 (3.78, 6.64)	6.29 (5.36, 7.39)	6.24 (5.25, 7.41)	5.91 (5.45, 6.41)
2009	6.68 (5.27, 8.43)	7.55 (6.50, 8.75)	6.27 (5.36, 7.32)	6.42 (5.92, 6.94)
2010	4.39 (3.43, 5.59)	7.36 (6.39, 8.46)	6.70 (5.92, 7.59)	5.54 (5.12, 5.99)
2011	6.14 (5.08, 7.41)	5.91 (5.17, 6.75)	6.50 (5.73, 7.36)	5.66 (5.24, 6.10)
2012	5.01 (3.96, 6.36)	6.03 (5.20, 6.97)	5.82 (5.01, 6.75)	5.61 (5.15, 6.11)
2013	4.29 (3.38, 5.44)	6.02 (5.14, 7.02)	5.95 (5.15, 6.83)	4.86 (4.47, 5.29)
2014	3.71 (2.83, 4.86)	4.86 (4.12, 5.72)	5.76 (5.01, 6.61)	4.89 (4.52, 5.30)
2015	5.93 (4.65, 7.54)	6.93 (6.04, 7.96)	5.79 (5.02, 6.65)	5.64 (5.23, 6.07)
2016	5.58 (4.31, 7.23)	6.31 (5.29, 7.50)	6.95 (5.90, 8.18)	6.35 (5.94, 6.79)
2017	6.94 (5.42, 8.84)	6.74 (5.50, 8.21)	6.84 (5.70, 8.19)	7.22 (6.74, 7.76)
2018	7.38 (5.61, 9.66)	7.39 (6.28, 8.69)	8.41 (7.17, 9.83)	7.85 (7.32, 8.40)

D) Waiting times too long to see the doctor

Year of Interview	Asian Individuals	Black Individuals	Hispanic/Latino Individuals	White Individuals
1999	1.87 (1.11, 3.15)	3.08 (2.50, 3.79)	3.80 (3.17, 4.55)	2.45 (2.23, 2.71)
2000	4.66 (3.05, 6.96)	3.66 (3.08, 4.33)	3.94 (3.33, 4.64)	3.70 (3.39, 4.03)
2001	5.02 (3.75, 6.68)	4.86 (4.18, 5.63)	5.54 (4.85, 6.33)	4.06 (3.71, 4.45)
2002	3.87 (2.79, 5.42)	4.85 (4.08, 5.77)	5.29 (4.50, 6.23)	3.71 (3.40, 4.04)
2003	3.85 (2.61, 5.62)	3.69 (3.12, 4.35)	6.36 (5.58, 7.22)	3.16 (2.86, 3.48)
2004	5.66 (4.08, 7.73)	5.39 (4.60, 6.30)	5.98 (5.20, 6.87)	4.17 (3.80, 4.59)
2005	6.21 (4.55, 8.42)	4.43 (3.70, 5.33)	6.94 (6.10, 7.83)	4.18 (3.83, 4.56)
2006	4.82 (3.66, 6.31)	4.37 (3.62, 5.25)	6.46 (5.57, 7.43)	3.67 (3.27, 4.10)
2007	3.62 (2.60, 5.03)	5.45 (4.58, 6.45)	7.22 (6.19, 8.39)	4.15 (3.75, 4.59)
2008	4.64 (3.52, 6.11)	6.54 (5.59, 7.63)	8.28 (7.10, 9.63)	4.30 (3.81, 4.84)
2009	7.86 (5.49, 11.19)	6.50 (5.55, 7.62)	8.47 (7.15, 10.06)	4.69 (4.27, 5.16)
2010	4.93 (3.88, 6.23)	6.43 (5.56, 7.46)	7.86 (7.06, 8.75)	3.68 (3.29, 4.09)
2011	5.77 (4.65, 7.17)	5.78 (5.00, 6.70)	7.95 (7.12, 8.86)	3.85 (3.54, 4.18)

© 2022 Caraballo C et al. *JAMA Health Forum.*

Year of Interview	Asian Individuals	Black Individuals	Hispanic/Latino Individuals	White Individuals
2012	4.52 (3.57, 5.71)	5.45 (4.67, 6.35)	6.81 (5.99, 7.74)	3.42 (3.11, 3.76)
2013	4.36 (3.39, 5.65)	4.91 (4.24, 5.67)	5.89 (5.03, 6.88)	2.86 (2.59, 3.15)
2014	3.83 (2.80, 5.25)	4.65 (4.03, 5.36)	5.81 (5.11, 6.58)	2.77 (2.51, 3.07)
2015	4.63 (3.52, 6.11)	5.30 (4.46, 6.30)	6.10 (5.29, 7.04)	3.07 (2.73, 3.44)
2016	5.29 (4.04, 6.93)	5.81 (4.69, 7.25)	6.99 (5.86, 8.29)	3.71 (3.38, 4.07)
2017	4.63 (3.37, 6.38)	4.48 (3.61, 5.54)	7.39 (6.27, 8.69)	3.98 (3.59, 4.42)
2018	4.47 (3.40, 5.87)	5.81 (4.81, 7.02)	7.65 (6.49, 8.99)	3.68 (3.34, 4.05)

E) Doctor's office not open when could get there

Year of Interview	Asian Individuals	Black Individuals	Hispanic/Latino Individuals	White Individuals
1999	2.14 (1.28, 3.64)	1.71 (1.33, 2.19)	2.04 (1.61, 2.58)	2.23 (2.01, 2.47)
2000	1.31 (0.79, 2.19)	1.86 (1.43, 2.39)	1.87 (1.48, 2.36)	2.53 (2.29, 2.79)
2001	2.01 (1.28, 3.17)	2.86 (2.23, 3.65)	2.59 (2.17, 3.07)	2.84 (2.59, 3.12)
2002	1.81 (1.12, 2.88)	1.93 (1.49, 2.50)	2.24 (1.74, 2.89)	2.34 (2.10, 2.61)
2003	1.25 (0.68, 2.27)	2.02 (1.51, 2.70)	1.84 (1.45, 2.32)	1.92 (1.70, 2.16)
2004	1.68 (0.98, 2.90)	2.07 (1.57, 2.71)	2.30 (1.86, 2.86)	2.59 (2.35, 2.84)
2005	2.87 (1.81, 4.51)	2.24 (1.73, 2.92)	2.44 (2.02, 2.96)	2.80 (2.52, 3.11)
2006	1.92 (1.23, 2.94)	2.35 (1.71, 3.23)	2.04 (1.58, 2.65)	2.77 (2.46, 3.12)
2007	1.46 (0.94, 2.25)	2.47 (1.97, 3.11)	2.95 (2.35, 3.72)	2.74 (2.44, 3.06)
2008	1.59 (0.96, 2.64)	2.84 (2.20, 3.65)	2.39 (1.87, 3.04)	3.52 (3.13, 3.96)
2009	2.83 (1.85, 4.32)	3.67 (3.00, 4.46)	3.08 (2.51, 3.79)	3.72 (3.28, 4.22)
2010	2.76 (1.97, 3.83)	3.12 (2.56, 3.80)	3.51 (2.91, 4.24)	2.91 (2.60, 3.25)
2011	2.15 (1.54, 2.95)	2.83 (2.30, 3.46)	3.27 (2.73, 3.92)	2.85 (2.59, 3.15)
2012	2.12 (1.35, 3.31)	2.29 (1.82, 2.89)	3.02 (2.50, 3.67)	2.85 (2.56, 3.18)
2013	2.28 (1.57, 3.30)	1.99 (1.56, 2.54)	2.46 (2.04, 2.96)	2.49 (2.24, 2.76)
2014	1.05 (0.64, 1.71)	2.13 (1.64, 2.77)	3.08 (2.63, 3.61)	2.28 (2.01, 2.58)
2015	2.01 (1.31, 3.04)	2.30 (1.81, 2.90)	2.69 (2.15, 3.38)	2.69 (2.38, 3.03)
2016	2.21 (1.39, 3.49)	2.30 (1.81, 2.90)	2.78 (2.17, 3.56)	2.60 (2.36, 2.87)
2017	2.02 (1.34, 3.02)	2.98 (2.30, 3.86)	2.90 (2.28, 3.67)	3.22 (2.92, 3.56)
2018	3.41 (2.37, 4.94)	3.15 (2.40, 4.14)	3.85 (3.05, 4.85)	3.81 (3.45, 4.20)

F) Did not have transportation

Year of Interview	Asian Individuals	Black Individuals	Hispanic/Latino Individuals	White Individuals
1999	1.70 (0.88, 3.21)	2.35 (1.84, 3.02)	1.71 (1.32, 2.21)	0.79 (0.68, 0.92)
2000	0.62 (0.28, 1.42)	2.44 (1.99, 3.00)	2.18 (1.73, 2.75)	1.06 (0.91, 1.22)
2001	0.70 (0.26, 1.86)	2.57 (2.10, 3.15)	2.28 (1.85, 2.82)	1.11 (0.96, 1.27)
2002	1.06 (0.63, 1.80)	2.88 (2.33, 3.53)	2.13 (1.62, 2.82)	1.02 (0.87, 1.20)
2003	0.91 (0.41, 2.10)	2.10 (1.68, 2.65)	1.58 (1.23, 2.02)	0.97 (0.84, 1.13)
2004	0.64 (0.28, 1.47)	3.30 (2.73, 3.98)	2.49 (2.05, 3.01)	1.25 (1.08, 1.44)
2005	1.30 (0.66, 2.64)	2.87 (2.33, 3.53)	2.49 (2.01, 3.07)	1.31 (1.14, 1.51)
2006	1.29 (0.75, 2.21)	3.37 (2.68, 4.24)	2.29 (1.79, 2.92)	1.27 (1.09, 1.47)

© 2022 Caraballo C et al. *JAMA Health Forum.*

Year of Interview	Asian Individuals	Black Individuals	Hispanic/Latino Individuals	White Individuals
2007	0.96 (0.46, 2.01)	2.81 (2.31, 3.41)	2.44 (1.90, 3.13)	1.31 (1.11, 1.54)
2008	1.50 (0.84, 2.65)	3.23 (2.58, 4.02)	2.96 (2.26, 3.83)	1.50 (1.26, 1.80)
2009	1.21 (0.76, 1.89)	4.33 (3.69, 5.08)	3.45 (2.70, 4.36)	1.57 (1.34, 1.83)
2010	1.24 (0.72, 2.13)	4.46 (3.72, 5.36)	3.11 (2.57, 3.75)	1.57 (1.35, 1.83)
2011	1.36 (0.90, 2.02)	4.14 (3.52, 4.84)	2.97 (2.48, 3.56)	1.56 (1.36, 1.79)
2012	0.89 (0.54, 1.44)	3.14 (2.59, 3.77)	2.66 (2.18, 3.23)	1.60 (1.40, 1.83)
2013	1.24 (0.74, 2.10)	2.84 (2.33, 3.45)	2.10 (1.74, 2.55)	1.43 (1.23, 1.65)
2014	1.16 (0.64, 2.09)	3.51 (2.91, 4.23)	2.28 (1.82, 2.86)	1.36 (1.17, 1.58)
2015	1.07 (0.71, 1.61)	3.66 (2.97, 4.49)	2.46 (1.96, 3.08)	1.39 (1.20, 1.61)
2016	0.88 (0.47, 1.63)	3.51 (2.84, 4.34)	2.24 (1.70, 2.99)	1.48 (1.28, 1.69)
2017	1.37 (0.74, 2.54)	3.02 (2.37, 3.84)	2.86 (2.19, 3.74)	1.48 (1.28, 1.71)
2018	2.07 (1.25, 3.43)	5.09 (4.11, 6.32)	2.71 (2.15, 3.41)	1.71 (1.48, 1.97)

© 2022 Caraballo C et al. *JAMA Health Forum.*

eTable 7. Change in the Adjusted Proportion of Individuals Reporting Delaying Care Because They Couldn't Get Through by Phone from 1999 to 2018, by Race and Ethnicity and Stratified by Sex and Income Level

	**Asian individuals**	**Black individuals**	**Hispanic/Latino individuals**	**White individuals**
	Percentage points (95% CI), p value	Percentage points (95% CI), p value	Percentage points (95% CI), p value	Percentage points (95% CI), p value
Absolute change in prevalence, 1999–2018				
Females	+1.48 (-0.49, +3.45), 0.14	+1.00 (-0.19, +2.19), 0.10	+1.67 (+0.46, +2.88), 0.01	+1.00 (+0.38, +1.63), 0.002
Males	-0.21 (-3.03, +2.61), 0.89	+2.06 (+0.63, +3.49), 0.01	+1.66 (+0.23, +3.09), 0.02	+0.78 (+0.31, +1.26), 0.001
Low-income	-0.94 (-4.71, +2.84), 0.63	+2.54 (+0.80, +4.29), 0.004	+2.25 (+0.63, +3.86), 0.01	+1.77 (+0.94, +2.60), <0.001
Middle/high-income	+1.37 (-0.49, +3.24), 0.15	+0.60 (-0.39, +1.59), 0.23	+1.17 (+0.02, +2.33), 0.05	+0.64 (+0.19, +1.10), 0.01
Difference with White, 1999				-
Females	-1.23 (-2.45, -0.01), 0.05	-0.54 (-1.25, +0.18), 0.14	-0.58 (-1.29, +0.13), 0.11	-
Males	+1.31 (-1.17, +3.80), 0.30	-0.81 (-1.36, -0.26), 0.004	-0.19 (-0.88, +0.50), 0.59	-
Low-income	+2.02 (-1.02, +5.07), 0.19	+0.05 (-0.77, +0.86), 0.91	-0.10 (-0.90, +0.70), 0.81	-
Middle/high-income	-0.82 (-2.24, +0.60), 0.26	-0.93 (-1.51, -0.35), 0.002	-0.50 (-1.21, +0.20), 0.16	-
Difference with White, 2018				-
Females	-0.75 (-2.42, +0.92), 0.38	-0.54 (-1.68, +0.60), 0.36	+0.09 (-1.07, +1.25), 0.88	-
Males	+0.32 (-1.09, +1.74), 0.66	+0.47 (-0.93, +1.87), 0.51	+0.68 (-0.66, +2.03), 0.32	-
Low-income	-0.69 (-3.07, +1.70), 0.57	+0.82 (-0.94, +2.57), 0.36	+0.38 (-1.25, +2.00), 0.65	-
Middle/high-income	-0.09 (-1.39, +1.21), 0.89	-0.97 (-1.90, -0.05), 0.04	+0.03 (-1.00, +1.05), 0.96	-
Change in difference with White, 1999–2018				-
Females	+0.48 (-1.59, +2.55), 0.65	-0.00 (-1.35, +1.34), 0.99	+0.67 (-0.69, +2.03), 0.34	-
Males	-0.99 (-3.85, +1.87), 0.50	+1.28 (-0.23, +2.79), 0.10	+0.88 (-0.64, +2.39), 0.26	-
Low-income	-2.71 (-6.58, +1.16), 0.17	+0.77 (-1.16, +2.70), 0.44	+0.47 (-1.34, +2.29), 0.61	-
Middle/high-income	+0.73 (-1.19, +2.65), 0.46	-0.04 (-1.13, +1.05), 0.94	+0.53 (-0.72, +1.77), 0.41	-

Data source is the National Health Interview Survey from years 1999 to 2018. For change in prevalence and change in difference: a positive sign (+) means the prevalence of this barrier (or its difference with White people) increased and a negative sign (-) means it decreased. Estimates were adjusted by age and US region.
Abbreviations: CI, confidence interval

© 2022 Caraballo C et al. *JAMA Health Forum.*

Supplemental eTable 8. Change in the Adjusted Proportion of Individuals Reporting Delaying Care Because They Couldn't Get an Appointment Soon Enough from 1999 to 2018, by Race and Ethnicity and Stratified by Sex and Income Level

	**Asian individuals**	**Black individuals**	**Hispanic/Latino individuals**	**White individuals**
	Percentage points (95% CI), p value	Percentage points (95% CI), p value	Percentage points (95% CI), p value	Percentage points (95% CI), p value
Absolute change in prevalence, 1999–2018				
Females	+4.35 (+0.94, +7.76), 0.01	+4.59 (+2.63, +6.55), <0.001	+5.42 (+3.28, +7.56), <0.001	+4.59 (+3.65, +5.53), <0.001
Males	+2.74 (-0.57, +6.06), 0.11	+4.55 (+2.71, +6.40), <0.001	+3.26 (+1.21, +5.32), 0.002	+3.57 (+2.82, +4.31), <0.001
Low-income	+6.30 (+0.87,11.73), 0.02	+5.38 (+3.19, +7.58), <0.001	+5.24 (+3.07, +7.41), <0.001	+5.62 (+4.39, +6.85), <0.001
Middle/high-income	+2.62 (-0.07, +5.32), 0.06	+3.84 (+2.12, +5.57), <0.001	+3.48 (+1.47, +5.48), <0.001	+3.66 (+2.97, +4.34), <0.001
Difference with White, 1999				-
Females	-1.37 (-3.26, +0.51), 0.15	-0.85 (-1.81, +0.11), 0.08	-0.09 (-1.19, +1.01), 0.87	-
Males	+1.52 (-0.88, +3.93), 0.22	-1.18 (-2.00, -0.35), 0.01	+0.69 (-0.44, +1.82), 0.23	-
Low-income	-0.39 (-3.40, +2.63), 0.80	-0.25 (-1.35, +0.84), 0.65	+0.19 (-0.86, +1.24), 0.73	-
Middle/high-income	+0.16 (-1.60, +1.91), 0.86	-1.14 (-1.98, -0.31), 0.01	+0.69 (-0.46, +1.85), 0.24	-
Difference with White, 2018				-
Females	-1.61 (-4.60, +1.38), 0.29	-0.85 (-2.80, +1.10), 0.39	+0.74 (-1.32, +2.80), 0.48	-
Males	+0.70 (-1.71, +3.10), 0.57	-0.19 (-2.00, +1.63), 0.84	+0.38 (-1.48, +2.25), 0.69	-
Low-income	+0.30 (-4.38, +4.97), 0.90	-0.49 (-2.75, +1.77), 0.67	-0.19 (-2.45, +2.07), 0.87	-
Middle/high-income	-0.88 (-3.03, +1.28), 0.42	-0.96 (-2.61, +0.70), 0.26	+0.51 (-1.26, +2.29), 0.57	-
Change in difference with White, 1999–2018				-
Females	-0.24 (-3.77, +3.30), 0.90	+0.00 (-2.17, +2.18), 0.99	+0.83 (-1.50, +3.17), 0.49	-
Males	-0.82 (-4.22, +2.58), 0.64	+0.99 (-1.00, +2.98), 0.33	-0.30 (-2.49, +1.88), 0.79	-
Low-income	+0.68 (-4.88, +6.24), 0.81	-0.23 (-2.75, +2.28), 0.86	-0.38 (-2.87, +2.11), 0.77	-
Middle/high-income	-1.03 (-3.81, +1.75), 0.47	+0.19 (-1.67, +2.04), 0.84	-0.18 (-2.30, +1.94), 0.87	-

Data source is the National Health Interview Survey from years 1999 to 2018. For change in prevalence and change in difference: a positive sign (+) means the prevalence of this barrier to timely medical care (or its difference with White people) increased and a negative sign (-) means it decreased. Estimates were adjusted by age and US region.
Abbreviations: CI, confidence interval

© 2022 Caraballo C et al. *JAMA Health Forum.*

eTable 9. Change in the Adjusted Proportion of Individuals Reporting Delaying Care Because They Had to Wait Too Long to See the Doctor from 1999 to 2018, by Race and Ethnicity and Stratified by Sex and Income Level

	**Asian individuals**	**Black individuals**	**Hispanic/Latino individuals**	**White individuals**
	Percentage points (95% CI), p value	Percentage points (95% CI), p value	Percentage points (95% CI), p value	Percentage points (95% CI), p value
Absolute change in prevalence, 1999–2018				
Females	+3.21 (+0.79, +5.63), 0.01	+2.82 (+1.05, +4.58), 0.002	+4.24 (+2.27, +6.21), <0.001	+1.63 (+1.03, +2.22), <0.001
Males	+1.92 (-0.54, +4.37), 0.13	+2.65 (+0.79, +4.51), 0.01	+3.48 (+1.39, +5.58), 0.001	+0.82 (+0.23, +1.41), 0.01
Low-income	+2.47 (-0.69, +5.63), 0.13	+4.25 (+2.05, +6.45), <0.001	+4.31 (+2.33, +6.30), <0.001	+2.03 (+1.00, +3.06), <0.001
Middle/high-income	+2.63 (+0.57, +4.70), 0.01	+1.64 (+0.15, +3.13), 0.03	+3.47 (+1.43, +5.52), <0.001	+1.02 (+0.55, +1.50), <0.001
Difference with White, 1999				-
Females	-0.71 (-2.20, +0.79), 0.35	+0.66 (-0.16, +1.48), 0.12	+1.57 (+0.42, +2.72), 0.01	-
Males	-0.49 (-2.37, +1.40), 0.61	+0.51 (-0.61, +1.62), 0.37	+1.09 (+0.03, +2.15), 0.04	-
Low-income	-1.06 (-3.13, +1.02), 0.32	+0.80 (-0.46, +2.06), 0.22	+0.88 (-0.24, +2.00), 0.125	-
Middle/high-income	-0.46 (-1.89, +0.96), 0.53	+0.23 (-0.53, +0.98), 0.55	+1.46 (+0.29, +2.63), 0.02	-
Difference with White, 2018				-
Females	+0.88 (-1.11, +2.87), 0.39	+1.85 (+0.18, +3.52), 0.03	+4.19 (+2.48, +5.90), <0.001	-
Males	+0.60 (-1.08, +2.29), 0.48	+2.33 (+0.73, +3.94), 0.004	+3.75 (+1.84, +5.65), <0.001	-
Low-income	-0.61 (-3.21, +1.98), 0.64	+3.02 (+0.94, +5.10), 0.004	+3.16 (+1.23, +5.10), 0.001	-
Middle/high-income	+1.15 (-0.42, +2.72), 0.15	+0.85 (-0.52, +2.21), 0.23	+3.91 (+2.17, +5.66), <0.001	-
Change in difference with White, 1999–2018				-
Females	+1.59 (-0.90, +4.08), 0.21	+1.19 (-0.67, +3.05), 0.21	+2.62 (+0.56, +4.68), 0.01	-
Males	+1.09 (-1.44, +3.62), 0.40	+1.82 (-0.13, +3.78), 0.07	+2.66 (+0.48, +4.83), 0.02	-
Low-income	+0.45 (-2.88, +3.77), 0.79	+2.22 (-0.21, +4.65), 0.07	+2.28 (+0.05, +4.52), 0.05	-
Middle/high-income	+1.61 (-0.51, +3.74), 0.14	+0.62 (-0.95, +2.18), 0.44	+2.45 (+0.35, +4.55), 0.02	-

Data source is the National Health Interview Survey from years 1999 to 2018. For change in prevalence and change in difference: a positive sign (+) means the prevalence of this barrier to timely medical care (or its difference with White people) increased and a negative sign (-) means it decreased. Estimates were adjusted by age and US region.
Abbreviations: CI, confidence interval

© 2022 Caraballo C et al. *JAMA Health Forum.*

eTable 10. Change in the Adjusted Proportion of Individuals Reporting Delaying Care Because the Doctor's Office Was Not Open When They Could Get There from 1999 to 2018, by Race and Ethnicity and Stratified by Sex and Income Level

	**Asian individuals**	**Black individuals**	**Hispanic/Latino individuals**	**White individuals**
	Percentage points (95% CI), p value	Percentage points (95% CI), p value	Percentage points (95% CI), p value	Percentage points (95% CI), p value
Absolute change in prevalence, 1999–2018				
Females	+2.20 (+0.01, +4.38), 0.05	+0.90 (-0.39, +2.18), 0.17	+1.87 (+0.51, +3.24), 0.01	+1.86 (+1.22, +2.50), <0.001
Males	+0.22 (-2.40, +2.84), 0.87	+2.23 (+0.81, +3.64), 0.002	+1.75 (+0.33, +3.18), 0.02	+1.31 (+0.70, +1.92), <0.001
Low-income	+0.34 (-3.01, +3.70), 0.84	+1.00 (-0.20, +2.20), 0.10	+1.67 (+0.05, +3.29), 0.04	+2.04 (+1.11, +2.98), <0.001
Middle/high-income	+1.68 (-0.31, +3.67), 0.10	+1.80 (+0.37, +3.23), 0.01	+1.93 (+0.69, +3.16), 0.002	+1.47 (+0.95, +1.99), <0.001
Difference with White, 1999				-
Females	-1.05 (-2.33, +0.23), 0.11	-0.43 (-1.12, +0.25), 0.22	-0.20 (-0.95, +0.55), 0.61	-
Males	+0.99 (-1.18, +3.15), 0.37	-0.79 (-1.46, -0.13), 0.02	-0.16 (-0.92, +0.61), 0.69	-
Low-income	+0.73 (-2.02, +3.49), 0.60	-0.31 (-1.10, +0.49), 0.45	+0.02 (-0.82, +0.86), 0.97	-
Middle/high-income	-0.41 (-1.73, +0.91), 0.54	-0.66 (-1.32, +0.00), 0.05	-0.32 (-1.01, +0.36), 0.36	-
Difference with White, 2018				-
Females	-0.71 (-2.59, +1.17), 0.46	-1.40 (-2.66, -0.13), 0.03	-0.19 (-1.49, +1.12), 0.78	-
Males	-0.10 (-1.69, +1.49), 0.90	+0.12 (-1.27, +1.51), 0.86	+0.29 (-1.06, +1.64), 0.68	-
Low-income	-0.97 (-3.10, +1.17), 0.38	-1.35 (-2.64, -0.05), 0.04	-0.36 (-2.03, +1.31), 0.68	-
Middle/high-income	-0.19 (-1.77, +1.39), 0.81	-0.33 (-1.69, +1.04), 0.64	+0.14 (-1.01, +1.29), 0.82	-
Change in difference with White, 1999–2018				-
Females	+0.34 (-1.94, +2.61), 0.77	-0.96 (-2.40, +0.47), 0.19	+0.01 (-1.50, +1.52), 0.99	-
Males	-1.09 (-3.78, +1.60), 0.43	+0.92 (-0.62, +2.45), 0.24	+0.44 (-1.11, +2.00), 0.58	-
Low-income	-1.70 (-5.19, +1.78), 0.34	-1.04 (-2.56, +0.48), 0.18	-0.37 (-2.24, +1.50), 0.70	-
Middle/high-income	+0.22 (-1.84, +2.27), 0.84	+0.33 (-1.19, +1.85), 0.67	+0.46 (-0.88, +1.80), 0.50	-

Data source is the National Health Interview Survey from years 1999 to 2018. For change in prevalence and change in difference: a positive sign (+) means the prevalence of this barrier to timely medical care (or its difference with White people) increased and a negative sign (-) means it decreased. Estimates were adjusted by age and US region.
Abbreviations: CI, confidence interval

© 2022 Caraballo C et al. *JAMA Health Forum.*

eTable 11. Change in the Adjusted Proportion of Individuals Reporting Delaying Care Because They Lacked Transportation from 1999 to 2018, by Race and Ethnicity and Stratified by Sex and Income Level

	**Asian individuals**	**Black individuals**	**Hispanic/Latino individuals**	**White individuals**
	Percentage points (95% CI), p value	Percentage points (95% CI), p value	Percentage points (95% CI), p value	Percentage points (95% CI), p value
Absolute change in prevalence, 1999–2018				
Females	+1.04 (-1.26, +3.34), 0.37	+2.44 (+0.98, +3.90), 0.001	+0.82 (-0.29, +1.92), 0.15	+1.00 (+0.60, +1.41), <0.001
Males	-0.32 (-2.77, +2.14), 0.80	+3.09 (+1.05, +5.13), 0.003	+1.25 (+0.17, +2.33), 0.02	+0.83 (+0.50, +1.16), <0.001
Low-income	+2.21 (-2.40, +6.83), 0.35	+4.86 (+2.37, +7.35), <0.001	+1.24 (-0.09, +2.57), 0.07	+3.29 (+2.37, +4.22), <0.001
Middle/high-income	-0.16 (-1.78, +1.45), 0.84	+1.29 (+0.01, +2.56), 0.05	+0.91 (-0.01, +1.83), 0.05	+0.31 (+0.09, +0.53), 0.01
Difference with White, 1999				-
Females	+0.54 (-1.02, +2.10), 0.50	+1.56 (+0.90, +2.21), <0.001	+1.34 (+0.63, +2.05), <0.001	-
Males	+1.21 (-0.73, +3.15), 0.22	+1.51 (+0.58, +2.45), 0.001	+0.45 (-0.20, +1.11), 0.17	-
Low-income	+0.24 (-2.45, +2.94), 0.86	+1.90 (+0.69, +3.11), 0.002	+0.41 (-0.49, +1.32), 0.37	-
Middle/high-income	+0.94 (-0.43, +2.31), 0.18	+0.55 (-0.04, +1.14), 0.07	+0.42 (-0.08, +0.92), 0.10	-
Difference with White, 2018				-
Females	+0.57 (-1.16, +2.31), 0.52	+2.99 (+1.63, +4.36), <0.001	+1.15 (+0.21, +2.09), 0.02	-
Males	+0.06 (-1.49, +1.61), 0.94	+3.78 (+1.93, +5.62), <0.001	+0.87 (-0.05, +1.79), 0.06	-
Low-income	-0.84 (-4.69, +3.02), 0.67	+3.47 (+1.10, +5.83), 0.004	-1.64 (-2.98, -0.30), 0.02	-
Middle/high-income	+0.47 (-0.42, +1.35), 0.30	+1.53 (+0.38, +2.68), 0.01	+1.03 (+0.23, +1.83), 0.02	-
Change in difference with White, 1999–2018				-
Females	+0.04 (-2.29, +2.37), 0.98	+1.44 (-0.07, +2.95), 0.06	-0.19 (-1.36, +0.99), 0.76	-
Males	-1.15 (-3.63, +1.33), 0.37	+2.26 (+0.19, +4.33), 0.03	+0.42 (-0.71, +1.55), 0.47	-
Low-income	-1.08 (-5.79, +3.63), 0.65	+1.57 (-1.09, +4.23), 0.25	-2.05 (-3.67, -0.43), 0.01	-
Middle/high-income	-0.47 (-2.10, +1.16), 0.57	+0.98 (-0.31, +2.27), 0.14	+0.61 (-0.34, +1.55), 0.21	-

Data source is the National Health Interview Survey from years 1999 to 2018. For change in prevalence and change in difference: a positive sign (+) means the prevalence of this barrier to timely medical care (or its difference with White people) increased and a negative sign (-) means it decreased. Estimates were adjusted by age and US region. Abbreviations: CI, confidence interval

© 2022 Caraballo C et al. *JAMA Health Forum.*

© 2022 Caraballo C et al. *JAMA Health Forum.*
